# Identifying Potential Geochemical and Microbial Impacts of Hydrogen Storage in a Deep Saline Aquifer

**DOI:** 10.1111/1758-2229.70076

**Published:** 2025-04-15

**Authors:** Kara A. Tinker, Winston Anthony, Meghan Brandi, Sam Flett, Christopher E. Bagwell, Chuck Smallwood, Ryan Davis, Djuna Gulliver

**Affiliations:** ^1^ National Energy Technology Laboratory Pittsburgh Pennsylvania USA; ^2^ NETL Support Contactor Pittsburgh Pennsylvania USA; ^3^ Pacific Northwest National Laboratory Richland Washington USA; ^4^ Sandia National Laboratories Albuquerque New Mexico USA

**Keywords:** geomicrobiology, hydrogen storage, subsurface microbiology

## Abstract

Hydrogen is valuable commodity and a promising energy carrier for variable energy production. Storage of hydrogen may occur through injection of hydrogen or a hydrogen/methane gas blend in subsurface reservoirs. However, the geochemical and biological reactions that may impact the stored hydrogen are not yet understood. Therefore, we collected samples from a deep storage aquifer located in the St. Peter Formation in southern Illinois. The reservoir material was primarily quartz with sulphur and iron deposits, while the major constituents of the fluid were chloride and sulphate. 16S rRNA gene amplicon sequencing revealed a low biomass microbial community that contained no obvious hydrogen‐consuming bacteria. Next, we enriched a field sample to increase the biomass and completed a metagenomic analysis, finding a low number of genes present that are associated with hydrogen consumption. Then, we completed a series of reactor experiments under reservoir conditions with 15% H_2_/85% CH_4_ gas simulating a short‐term hydrogen storage, high withdrawal scenario. We found minimal changes in the geochemistry or microbiology for the reactor experiments. This work suggests that short‐term storage may be highly successful, although significant additional work needs to be completed in order to accurately evaluate the risks associated with long‐term hydrogen storage scenarios. It is essential we continue to expand our understanding of the dynamics present in saline aquifers and provide new insights into how hydrogen storage may impact underground geological storage environments.

## Graphical abstract

Subsurface Hydrogen Assessment, Storage, and Technology Acceleration (SHASTA) cartoon showing potential microbial activity that may impact subsurface hydrogen storage.

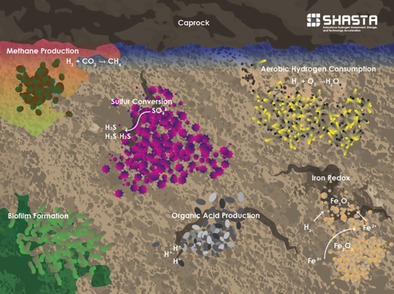

## 1 Introduction

Hydrogen can be produced from multiple sources and is a valuable domestic commodity. Additionally, it can have an important role a carrier of variable energy production due to its high energy density (Goodman et al. 2022; Mazloomi and Gomes 2012). To utilise hydrogen as an energy carrier, we must develop large‐scale hydrogen storage facilities that allow for the decoupling of energy production and demand (Moradi and Groth 2019; Zivar et al. 2021). In the United States, the primary approach is to blend hydrogen with methane in order to leverage current methane storage infrastructure. Currently, the most promising large‐scale storage sites are depleted hydrocarbon reservoirs, saline aquifers, salt caverns, and hard rock caverns (Goodman et al. 2022; Moradi and Groth 2019; Zivar et al. 2021; Dopffel et al. 2021). However, each of these geologic formations has unique properties and challenges (Goodman et al. 2022; Zivar et al. 2021; Dopffel et al. 2021), necessitating the study of each type of reservoir for the development of large‐scale hydrogen or hydrogen/methane storage technologies.

Saline aquifers have been used for decades for natural gas storage; however, they have not been thoroughly evaluated as potential hydrogen storage locations (Goodman et al. 2022; Dopffel et al. 2021). Saline aquifers are found in most sedimentary basins and have a high storage capacity (Goodman et al. 2022; Sainz‐Garcia et al. 2017; Al‐Shafi et al. 2023). Recent modelling work has demonstrated feasibility, as saline aquifers can have promising recovery ratios, especially in instances when caprock is present in the reservoir (Sainz‐Garcia et al. 2017). However, the long‐term success of hydrogen or hydrogen blend storage in saline aquifers or other geological environments is highly dependent on both the resident microbial community and the unique geological properties present in the environment (Dopffel et al. 2021; Bassani et al. 2023; Bellini et al. 2024). For example, corrosion, biofouling, and souring events may be more likely to occur if the reservoir is mesophilic or contains high amounts of carbonate and sulphate minerals, as these conditions would support more rapid microbial activity and growth (Dopffel et al. 2021; Gieg et al. 2011; Ebigbo et al. 2013; Liang et al. 2014; Mand et al. 2014; Booker et al. 2017; Loto 2017; Lahme et al. 2018; Gregory et al. 2019; Kotu et al. 2019; Tang et al. 2019; Tinker et al. 2022). In addition, subsurface microorganisms present in saline aquifers could induce gas mixture changes and/or changes in reservoir properties when exposed to hydrogen (Dopffel et al. 2021; Ebigbo et al. 2013; Liang et al. 2014; Kotelnikova 2002; McGenity 2010; Grein et al. 2013; An et al. 2017; Cliffe et al. 2020; Tyne et al. 2021). Methanogenesis, acetogenesis, and sulphate reduction are the most likely microbial hydrogen‐consuming reactions; although other reactions, including sulphur reduction, iron reduction, denitrification, and aerobic H_2_ oxidation, are also of concern depending on reservoir conditions and geochemistry (Dopffel et al. 2021; Ebigbo et al. 2013; Liang et al. 2014; Kotelnikova 2002; McGenity 2010; Grein et al. 2013; An et al. 2017; Cliffe et al. 2020; Tyne et al. 2021; Aftab et al. 2022; Jafari Raad et al. 2022).

To explore the potential impacts of hydrogen storage, we collected field samples from a deep saline aquifer undergoing development for hydrogen gas storage. We characterised the physical, chemical, and microbial compositions of the field samples using scanning electron microscopy (SEM), geochemical analysis, qPCR, and 16S rRNA gene amplicon sequencing. Next, we utilised two complementary approaches to investigate potential risks and challenges at our field site. First, we completed metagenomic sequencing on an enriched field sample to identify potential metabolic activity that may occur in our field site. Second, we completed a series of reactor experiments in order to simulate hydrogen/methane storage conditions in a short‐term storage, high hydrogen‐withdrawal scenario. This allowed us to capture changes in geochemical, microbiological, and gas measurements at various time points throughout the storage process. To our knowledge, this work represents the first published reactor experiments for hydrogen/methane storage in a subsurface saline aquifer. Given the likely increase need for hydrogen storage and blended hydrogen/methane storage in the future, these findings provide an important starting point for understanding microbial and geochemical dynamics present in saline aquifers and illustrate potential challenges that may occur while monitoring and storing hydrogen gas in subsurface environments.

## 2 Experimental Procedures

### 2.1 Field Sampling

Samples were collected during a drill stem test (DST) completed in July 2021 located within a deep saline aquifer in Southern Illinois. A DST is an opportunity to collect in situ biological samples from the subsurface with high subsurface fluid flow rates that reduce the risk of contamination from the wellbore/operating equipment (Lyons 2010). A small section of core sample provided during the drilling process was immediately frozen for preservation. Produced fluid samples were collected from the St. Peter Formation at a depth of 930–1042 m in sanitised containers. To maintain the microbial diversity of the reservoir, a portion of the water phase was collected in sterile 1 L nalgene bottles and immediately placed on dry ice. This preservation method has been previously reported and utilised in the literature (Tinker et al. 2022, 2020; Struchtemeyer and Elshahed 2012; Murali Mohan, Hartsock, Bibby, et al. 2013; Murali Mohan, Hartsock, Hammack, et al. 2013; Cluff et al. 2014; Mohan et al. 2014; Lipus et al. 2017, 2018, 2019) and was selected to reduce the extreme microbial structure change that can occur during the storage of thawed material. Upon arrival in the lab, the frozen field samples were stored at −20°C until thawed at 5°C before immediately processing or use in reactor experiments. The remaining field sample was passed through a glass wool cartridge under gravity followed by a 0.45 μm inline filter (EnviroTech, Salt Lake City, Utah) in a closed loop to minimise oxygen exposure. The filtered water was measured for pH on‐site using a YSI multimeter (Xylem, Yellow Springs, OH) before being titrated for alkalinity using a Hach digital titrator (Hach, Loveland, CO) and discarded on‐site.

### 2.2 Reactor Conditions for Gas Storage Simulations

Three sets of reactor experiments were completed. The initial reactors were conducted in 150 mL SilcoTek treated steel reactors run at the field sample reservoir conditions (47°C and ~1800 psi) with either 100% N_2_ or a 15% H_2_/85% CH_4_ gas blend and 100 mL of produced fluid. Abiotic (filter sterilised) and biotic (unfiltered) treatments were completed, and the experiments were run in duplicate, with individual reactors sacrificed for analysis after 1 day, 2 days, and 3 days. The follow‐up reactor series contained approximately 90 mL of unfiltered produced fluid and 10 g of crushed sediment from the core. The experiment was run in duplicate, with individual reactors sacrificed for analysis after 1, 3, 7 and 21 days. Fluid for abiotic treatments was prepared by filter sterilisation with 0.2 μm polyethersulfone membrane filters (Qiagen, Hilden, Germany).

When sacrificing the reactors, gas was collected into 1 L tedlar bags (Fisher Scientific, Waltham, MA). Field and reactor sample fluids were filtered through 0.2 μm polyethersulfone membrane filters (Qiagen, Hilden, Germany) to collect microbial biomass and then frozen at −20°C. A 15 mL volume of the resulting filtrate was stored for ion chromatography (IC) measurement and a 30 mL volume of the filtrate was acidified to 2% by volume using hydrochloric acid for inductively coupled plasma optical emission spectroscopy (ICP‐OES); both samples were stored frozen at −20°C until chemically analysed. Any remaining sediment sample from the reactors was stored frozen until analysis by SEM. Note that short‐chain organic acid concentrations for the initial two reactor sets (100% N_2_, without sediment and 85% CH4/15%H_2_, without sediment) were recorded but not reported in Table 1, as they were either undetectable (acetate, propionate, butyrate, and succinate) or minimal (< 4 mg/L oxalate and < 5 mg/L formate).

**TABLE 1:** Geochemical measurements for the field sample and reactors at each time point.

			B^a^	Ba^a^	Ca^a^	Fe^a^	K^a^	Li^a^	Mg^a^	Mn^a^	Na^a^	P^a^	Sr^a^	Cl^b^	SO_4_ ^b^	Br^b^	PO_4_ ^b^
	Field sample	DST fluid	3.25	0.28	2730	BDL	215	5.97	808	1.03	11,480	0.44	70	30,857	3280	BDL	BDL
100% N_2_, without sediment	Abiotic	Day 1	3.82	0.32	2681	0.30	262	7.32	946	1.04	13,176	0.11	78	22,757	915	93	BDL
		Day 2	3.62	0.29	2711	0.46	260	6.88	927	0.95	13,106	BDL	77	30,149	1212	121	BDL
		Day 3	3.76	0.31	2716	0.33	264	6.98	947	0.93	13,026	BDL	78	29,972	1207	120	BDL
	Abiotic replicate	Day 1	3.75	0.30	2640	0.36	264	7.22	944	1.03	12,936	BDL	79	29,936	1210	121	BDL
		Day 2	3.76	0.35	2619	0.35	264	6.90	942	1.08	12,536	BDL	77	29,342	1181	118	BDL
		Day 3	3.71	0.30	2732	0.54	270	7.01	945	1.27	12,926	BDL	80	29,956	1208	120	BDL
	Biotic	Day 1	2.96	0.25	2696	1.07	212	5.48	740	1.07	12,466	BDL	69	27,974	2195	102	BDL
		Day 2	2.93	0.24	2625	0.74	211	5.41	741	0.74	12,756	BDL	68	27,486	1950	101	BDL
		Day 3	2.88	0.26	2549	0.88	211	5.37	733	0.88	12,526	BDL	69	27,482	1830	100	BDL
	Biotic replicate	Day 1	2.82	0.23	2602	0.67	204	5.21	711	0.67	12,276	BDL	67	26,700	2064	98	BDL
		Day 2	2.98	0.25	2529	1.46	218	5.62	747	1.46	12,376	BDL	69	27,288	1181	100	BDL
		Day 3	3.36	0.26	2531	0.77	214	5.37	738	0.77	12,196	BDL	69	27,531	1894	101	BDL
Without sediment	Abiotic	Day 1	2.89	0.31	3129	0.35	253	6.84	916	1.05	12,073	BDL	78	31,736	2380	142	BDL
		Day 2	2.68	0.26	2913	0.32	244	6.23	861	1.14	11,073	BDL	73	31,056	2339	135	BDL
		Day 3	2.99	0.30	3087	0.25	269	6.81	911	1.11	11,683	BDL	77	31,078	2297	135	BDL
	Abiotic replicate	Day 1	2.62	0.28	3062	0.36	238	6.05	909	1.07	11,873	BDL	74	31,024	2316	135	BDL
		Day 2	3.01	0.29	3045	1.56	269	7.05	924	1.12	11,823	BDL	81	31,869	2283	139	BDL
		Day 3	2.72	0.28	3125	0.36	256	6.40	933	0.96	12,093	BDL	73	30,579	2269	131	BDL
	Biotic	Day 1	2.73	0.35	2997	0.21	255	6.30	916	1.12	11,813	BDL	77	31,894	2407	146	BDL
		Day 2	3.01	0.29	3039	0.30	276	7.01	933	1.62	11,763	BDL	79	32,270	2238	142	BDL
		Day 3	2.85	0.30	3037	0.44	256	6.62	904	1.23	11,653	BDL	75	30,447	2266	132	BDL
	Biotic replicate	Day 1	2.78	0.30	3062	0.21	250	6.32	893	1.06	11,643	BDL	75	30,722	2320	140	BDL
		Day 2	2.89	0.28	3111	0.25	259	6.59	950	1.54	12,103	BDL	76	31,902	2208	139	BDL
		Day 3	3.03	0.30	3145	0.39	266	6.78	926	1.05	11,923	BDL	77	31,052	2313	134	BDL
With sediment	Biotic	Day 1	2.98	0.34	2351	BDL	201	5.71	775	1.02	10,650	0.45	64	30,917	2217	BDL	BDL
		Day 2	3.45	0.34	2568	BDL	232	6.38	871	1.37	11,960	0.43	72	30,480	2244	BDL	BDL
		Day 7	3.24	0.35	2700	BDL	228	6.18	868	1.16	11,820	BDL	72	26,523	2475	BDL	BDL
		Day 21	3.14	1.30	2491	BDL	222	6.37	865	2.91	12,080	0.39	71	33,760	2689	BDL	BDL
	Biotic replicate	Day 1	3.22	0.32	2591	BDL	217	6.14	822	1.23	11,290	0.42	69	33,930	2128	BDL	BDL
		Day 2	3.28	0.31	2484	BDL	217	5.94	828	1.05	11,200	0.44	68	28,360	1979	BDL	BDL
		Day 7	2.96	0.38	2332	BDL	201	5.52	769	1.07	10,540	BDL	63	23,973	2876	BDL	BDL
		Day 21	3.38	0.34	2629	102	227	6.39	861	1.35	11,950	0.53	72	29,860	2399	BDL	BDL

*Note:* All units are in mg/L.

Abbreviation: BDL, below detection limit.

^a^ Measured via ICP.

^b^ Measured via IC.

### 2.3 SEM, Geochemical Analysis and Gas Chromatography

Crushed sediment core reserved from the field sample as well as the Day 1 and Day 21 follow‐up reactors was analysed with SEM. Samples were air‐dried, mounted onto a standard aluminium pin mount stub with carbon conductive tabs, sputter coated with Au‐Pd at approximately 10 nm thickness, and imaged via a back‐scattered electron detector (BSED) on an FEI Quanta 600F SEM equipped with Oxford's INCAx‐act detector for energy dispersive spectroscopy (EDS) compositional analysis. BSED imaging across the samples at a range of magnifications was used to highlight any differences in elemental composition by grayscale value. Imaging was accompanied by EDS false colour element maps and point spectra. All SEM analyses were performed under high vacuum conditions with a beam voltage of 15 keV and a spot size of 3.0um.

Geochemical analyses were completed as previously described (Tinker et al. 2022) using a Thermo Fisher (Thermo Fisher, Waltham, MA) ICS‐5000+ with AS11‐HC column for anion quantification and CS16 column for cation quantification. Trace metals were analysed using the U.S. Environmental Protection Agency (EPA) Method 6010D with ICP‐OES on an Optima 7300 DV (Perkin Elmer, Waltham, MA). Finally, gas samples were collected in a tedlar bag and immediately analysed to measure H_2_, CO_2_, and/or CH_4_ concentrations using a PerkinElmer Clarus 600 gas chromatograph (PerkinElmer, Waltham, MA) with a flame ionisation detector (FID) and a thermal conductivity detector (TCD).

### 2.4 DNA Extraction and qPCR

DNA was extracted from 0.2 μm filters using the DNeasy Powersoil Pro Kit (Qiagen, Hilden, Germany) before eluting the DNA in 100 μL of buffer. Kit blanks were concurrently extracted to confirm that no contamination occurred. Abiotic samples were also extracted in order to confirm that no biomass was present. Microbial biomass load in produced water samples was measured using qPCR as previously described (Tinker et al. 2020) using Universal 16S rRNA gene primers (F: GTGSTGCAYGGYTGTCGTCA ( *E. coli* position 1048–1067); R: ACGTCRTCCMCACCTTCCTC ( *E. coli* position 1175–1194)) designed by Maeda et al. (2003) and standard curves generated using gBlocks Gene Fragments (Integrated DNA Technologies, Coralville, IA) (Data S1). Extracted DNA was amplified using universal primers targeting the V4 region of the 16S rRNA gene, as previously described (Caporaso et al. 2011, 2012). PCR amplicons were cleaned using SPRIselect beads (Beckman Coulter, Pasadena, CA), visualised using a Bioanalyzer (Agilent, Santa Clara, CA), and quantified using a Qubit (Life Technologies, Carlsbad, CA). Negative controls were also amplified, visualised, and quantified to confirm that no contamination occurred. Any kit blanks and negative control samples with measurable amplification were sequenced concurrently with experiment samples and later utilised in downstream quality control filtering. Purified 16S rRNA gene amplicon libraries were pooled (with 8 replicates per sample), diluted to a concentration of 2 nM, and denatured using fresh 0.2 M NaOH. Libraries were further diluted according to the manufacturer's instructions and sequenced on an Illumina Miseq (Illumina, San Diego, CA) using a 300 cycle V2 Nano kit.

### 2.5 Shotgun Metagenomic Generation, Sequence Processing, and Data Analysis

Extracted DNA was amplified for metagenomic sequencing using the Illumina Nextera XT kit (Illumina, San Diego, CA). However, library preparation was unsuccessful due to the quality and amount of DNA available. Therefore, we removed a 1 L nalgene bottle of produced water from long‐term storage at −20°C and placed it at 4°C for 42 days to accumulate a higher biomass. No additional nutrients were added to the produced water sample. We collected microbial cells on a Sterivex (MilliporeSigma, Burlington, MA) filter and extracted DNA using the DNeasy PowerWater Sterivex Kit (Qiagen, Hilden, Germany). The resulting DNA was sent to Genewiz (Azenta, South Plainfield, NJ) for whole genome metagenomics. Genome binning was conducted using the metaGEM sequence pipeline with default settings. Briefly, low‐quality reads were removed using fastp (Chen et al. 2018) before contig assembly using megahit (Li et al. 2015). Draft genome bins from all tools were refined and reassembled using metaWRAP (Uritskiy et al. 2018) and taxonomy was identified using GTDB‐tK (Chaumeil et al. 2020). On Kbase (Arkin et al. 2018), DRAM (Shaffer et al. 2020) was used to annotate genome bins with the KOfam database (Aramaki et al. 2020) as well as determine the KEGG (Kanehisa and Goto 2000; Kanehisa et al. 2012) pathway coverage.

### 2.6 16S rRNA Gene Amplicon Library Preparation, Sequencing and Analysis

Extracted DNA was amplified using universal primers targeting the V4 region of the 16S rRNA gene, as previously described (Caporaso et al. 2011, 2012). PCR amplicons were cleaned using SPRIselect beads (Beckman Coulter, Pasadena, CA), visualised using a Bioanalyzer (Agilent, Santa Clara, CA), and quantified using a Qubit (Life Technologies, Carlsbad, CA). Negative controls were also amplified, visualised, and quantified to confirm that no contamination occurred. Any kit blanks and negative control samples with measurable amplification were sequenced concurrently with experiment samples and later utilised in downstream quality control filtering. Purified 16S rRNA gene amplicon libraries were pooled (with 8 replicates per sample), diluted to a concentration of 2 nM, and denatured using fresh 0.2 M NaOH. Libraries were further diluted according to the manufacturer's instructions and sequenced on an Illumina Miseq (Illumina, San Diego, CA) using a 300 cycle V2 Nano kit.

16S rRNA gene amplicon sequences were analysed using QIIME2 version 2022.11 (Bolyen et al. 2019) as previously described (Tinker et al. 2020). In brief, with all settings modifications listed: Sequences were imported as EMPSingleEndSequences, demultiplexed using the demux emp‐single command, and processed for quality control using DADA2 (Callahan et al. 2016) with a truncation length of 250 base pairs. The classify‐sklearn (Pedregosa et al. 2011) command was used to classify representative sequences using a pre‐trained Naive Bayes classifier trained on Silva 138 with 99% OTUs (Pruesse et al. 2007; Quast et al. 2012) from the 515F/806R region, and sequences identified as chloroplast or mitochondria were filtered from the data. This effort resulted in a total of 1,023,635 raw sequences, with 174,391 remaining after quality control filtering (Table S1).

**FIGURE 1:**
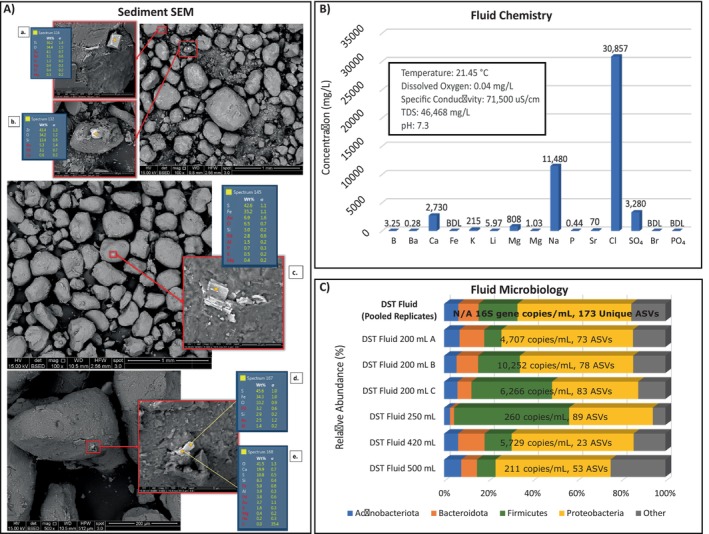
Field sample characterisation. (A) Sediment SEM. Backscattered SEM images of (a.) a titanium oxide mineral (likely anatase), (b.) a zircon, (c.) pyrite, (d.) pyrite, and (e.) possible gypsum within quartz sandstone grains, as well as identifying EDS point spectra results. Au‐Pd conductive coating applied as sample preparation. (B) Fluid Chemistry. Field measurement, cations, and anions for the DST fluid sample. Cations were measured with ICP and anions were measured with IC. All measurements are in mg/L; “BDL” stands for below detection limit. (C) Fluid Microbiology. Relative abundance of major phyla in DST fluid samples (pooled and individual replicates). 16S gene copies/mL and the total number of unique ASVs for each sample are also displayed.

Data generated by Qiime2 was imported into R for further quality control filtering (R Core Team 2013). First, any individual sample with < 1000 sequences remaining after quality control filtering was discarded (Table S2). Data were normalised, with no subsampling, and used to build bar and bubble plots (Figures 1C and 3). The Vegan package (Oksanen et al. 2012) was used to complete non‐metric multidimensional scaling (NMDS) analysis and construct an ordination plot with *k* = 3 dimensions using sequencing libraries subsampled to the lowest sequence depth (1046 sequences per sample). Any individual samples that clustered with kit blanks or negative controls were discarded (Table S1). Technical replicates from the remaining samples were then pooled and analysed in R (R Core Team 2013). Rarefaction curves were used to confirm that we appropriately captured species richness for all the field and reactor samples (Figure S1, Table S3).

After quality control was complete, the number of observed amplicon sequence variants (ASVs) and Pielou's evenness were calculated in Base R (R Core Team 2013) for each sample. The Vegan package (Oksanen et al. 2012) was used to calculate the Chao1 index, Shannon index, and Bray–Curtis dissimilarity values as well as complete NMDS analyses, fit environmental parameters onto the generated ordination plot, and complete the Mantel test. All NMDS plots were constructed with *k* = 3 dimensions with visualisation of the first two dimensions. NMDS and the Mantel test were completed using Bray–Curtis distance measurements calculated after sequencing libraries were subsampled to the lowest sequence depth (1046 sequences per sample) represented across all samples (Tables S1 and S2). The Mantel test also relied on the Euclidean distance of the geochemical data, which was calculated in Base R. The statistical significance of the Mantel test and the environmental vectors was based on 999 permutations of the data.

## 3 Results

### 3.1 Characterisation of Field Samples From a Deep Saline Aquifer

Core and fluid samples were collected during a DST completed in July 2021 located within a deep saline aquifer in Southern Illinois. Sediment from the crushed core was evaluated using SEM‐EDS to determine the geologic composition. Sediments were found to be primarily composed of quartz sandstone with some occurrences of pyrite (FeS2), zircon (ZrSiO4), and titanium oxide minerals (TiO_2_, presumed anatase) (Figure 1A). Minor amounts of possible gypsum (CaSO4) and trace amounts of Al, Ca, P, Mg, Na, K and Mn were detected (Figure 1A). The pH of the DST reservoir fluid was 7.3 with a total dissolved solids (TDS) concentration of 46,468 mg/L (Figure 1B). The major anions were chloride and sulphate, at 30,857 and 3280 mg/L (Figure 1B). The major cations, calcium and sodium, were present at concentrations of 2730 and 11,480 mg/L, respectively (Figure 1B). Minimal amounts of boron, barium, potassium, lithium, magnesium, manganese, phosphorus and strontium were also detected (Figure 1B). No iron, bromide or phosphate was measured in the field sample (Figure 1B).

The microbial load of the DST reservoir water was variable between sample replicates, ranging from 211 to 10,252 16S rRNA gene copies/mL of sample (Figure 1C, Table S2). One potential reason for this variation is that the fluid has low biomass, thus making it challenging to recover a homogeneous distribution of cells in a fluid sample. Despite the variation in microbial load, we found that the microbial community composition of each DST reservoir sample replicate was highly congruent and primarily composed of halophilic anaerobic organisms that have previously been associated with subsurface environments (Figure 1C). We also identified several aerobic microbial taxa in the DNA sample taken directly from the field. Although this is somewhat surprising, there is increasing evidence that aerobic microbial taxa are common in subsurface environments, especially in groundwater systems (An et al. 2013; Rollick 2014; Ridley and Voordouw 2018; Ruff et al. 2023). Overall, we found that Proteobacteria was the dominant phylum, representing an average relative abundance of 57.27% across all samples (Figure 1C). Firmicutes and Bacteroidota were prevalent, with average relative abundances of 24.11% and 11.02% across all samples (Figure 1C). Members of the Actinobacteria phylum were present in all samples but only represented 1.11% to 10.92% relative abundance in any one sample (Figure 1C). Notably, we identified no Archaea in any of the DST fluid or reactor samples. However, this may be due to limitations with the universal 515F/806R primers (Bahram et al. 2019).

### 3.2 Functional Potential in an Enriched Field Sample

**FIGURE 2:**
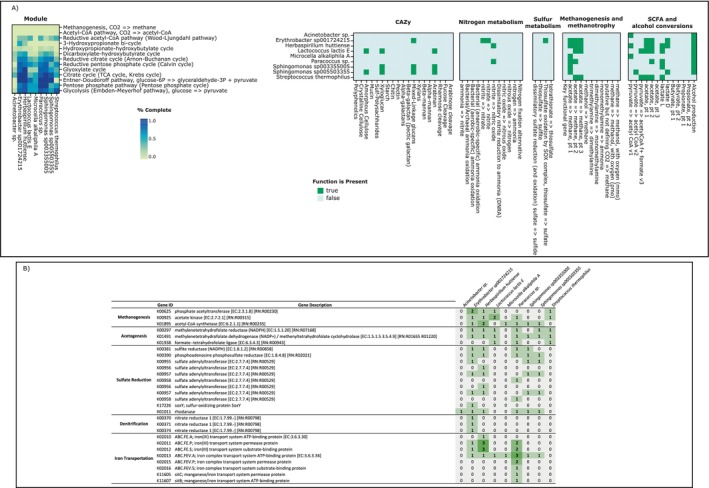
Metabolic potential in an enriched saline aquifer sample. (A) Modified DRAM output displaying relevant pathway and functional coverage for the recovered MAGs. Full DRAM output is displayed in Figure S2. (B) Table showing the KO identifier, gene description, and presence/absence information for genes associated with methanogenesis, acetogenesis, sulphate reduction, denitrification, and iron transportation. Note that only genes with a presence in at least one MAG are displayed. A complete table with all genes associated with these processes is displayed in Table S5.

**FIGURE 3:**
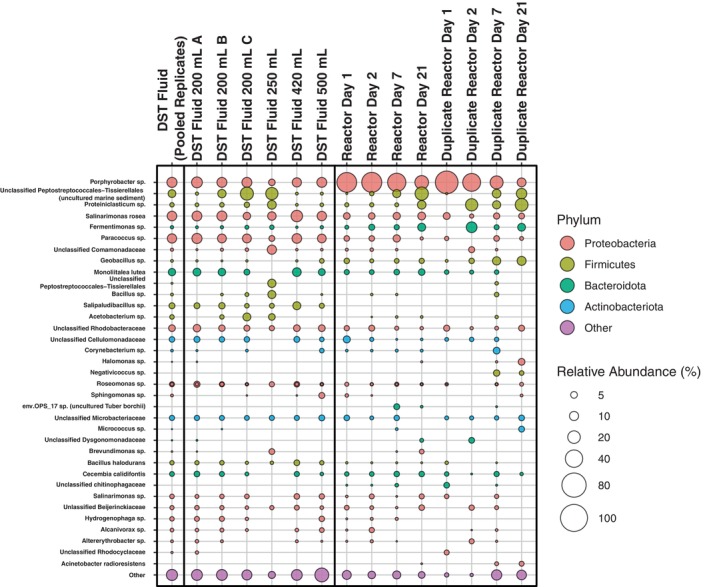
DST and reactor microbial community. Bubble plot showing the relative abundance of taxa found within the experimental samples. Any taxa that comprised > 2.5% of one sample are shown. Low abundance taxa were combined and shown as Other.

Initially, we were interested in examining the functional potential present in the DST reservoir fluid. However, low biomass made it impossible to prepare a metagenomic library for sequencing. Therefore, we opted to complete a metagenomic analysis on an enriched field sample. For this enrichment, we did not add any additional nutrients; rather, we simply allowed the material to increase in biomass while stored at 4°C over 42 days. We assembled 9 metagenome‐assembled genomes (MAGs): 5 high quality (> 85% completeness, < 3% contamination) and 4 medium quality (> 55% completeness, < 3% contamination), with 7 classified to a species level taxonomic assignment (Figure 2, Table S4). These MAGs were identified as: *Acinetobacter sp., Erythrobacter sp001724215, Herbaspirillum huttiense, Lactococcus lactis E, Microcella alkaliphila A, Paracoccus sp., Sphingomonas sp003355005, Sphingomonas sp005503355* and *Streptococcus thermophilus*. We utilised the taxonomic data obtained from 16S rRNA gene amplicon sequencing (Figure 3) to determine the abundance of these MAGS in our field sample and reactor experiments. *Erythrobacter sp001724215, H. huttiense*, and *Microcella alkaliphila A* were not present in our 16S rRNA gene amplicon data. However, across our field and reactor samples, we found that *Acinetobacter sp., L. lactis, Paracoccus sp., Sphingomonas sp*., and *Streptococcus sp*. were represented at a maximum relative abundance of 2.49%, 0.81%, 12.56%, 3.78% and 0.55%, respectively.

Microbial consumption of hydrogen is a major concern in underground hydrogen storage environments. Therefore, we examined the KEGG pathway and functional coverage for hydrogen‐consuming reactions in the 9 MAGs (Figure 2). Methanogenesis and acetogenesis are the most likely microbial hydrogen‐consuming reactions. However, these reactions are unlikely to occur in the enrichment, as none of the MAGs contained any KEGG pathway modules associated with methanogenesis, and the module for the Wood‐Ljungdahl pathway was either partially complete (< 50% for 7 MAGs) or absent across all MAGs (Figure 2). Another common hydrogen‐consuming reaction is sulphate reduction, although minimal genes associated with assimilatory and dissimilatory sulphate reduction were present across the MAGs from the enrichment (Figure 2). Interestingly, 6 of the 9 MAGs contained the genes for rhodanese (Figure 2B), which is associated with the conversion of thiosulphate to adenyl sulphate and is an essential component of thiosulphate reduction. This is notable, as thiosulphate reduction is increasingly thought to be a common form of sulphate reduction in subsurface environments (Liang et al. 2014; Booker et al. 2017; Tinker et al. 2022; Choudhary et al. 2015). Less common hydrogen‐consuming reactions are iron reduction and denitrification. Although there is no iron reduction KEGG module, we examined all the KO identifiers associated with iron transport and found minimal genes present (Figure 2B). We also utilised FeGenie (Garber et al. 2020), a comprehensive tool for the identification of iron genes, and found that none of the MAGs contained any genes associated with iron reduction. Finally, we found few genes present associated with denitrification (Figure 2), suggesting it is unlikely to occur in our enriched field sample.

### 3.3 Hydrogen Storage Reactor Experiments

We completed a series of reactor experiments with the goal of understanding the impact of short‐term hydrogen blend storage in a deep saline aquifer. For the initial experiments, we incubated produced fluid from our field sampling effort at reservoir conditions (47°C and ~1800 psi) with either a 100% N_2_ or 15% H_2_/85% CH_4_ gas blend. The 100% N_2_ acted as a control, as the inert headspace allowed us to identify geochemical changes that may occur without any exposure to injected H_2_/CH_4_ gas. In contrast, the 15% H_2_/85% CH_4_ gas blend was chosen because it simulates a blended hydrogen environment and utilises a hydrogen fraction within estimated potential storage limits for the United States (Lackey et al. 2023). Both of these initial reactor series were run in duplicate with abiotic (filter sterilised) and biotic (unfiltered) treatments, with individual reactors sacrificed for analysis after 1, 2 and 3 days.

No gas headspace changes were recorded for the control 100% N_2_ reactor. However, we found a rapid decrease in the overall percent hydrogen in the headspace for the 15% H_2_/85% CH_4_ gas blend reactors (Table S6). This value ranged from 10.63% to 11.52% for the abiotic reactors and 9.71%–12.5% for the biotic reactors, with replicate 1 day abiotic reactor being an outlier with complete hydrogen consumption (Table S6). We were also interested in measuring how the geochemical trends present in the reactor series compared to the starting reservoir fluid (Table 1). The majority of the ions did not display significant changes for either reactor series, although sulphate concentrations were both highly variable and lower across all reactors (abiotic and biotic; 100% N_2_ and 15% H_2_/85% CH_4_), ranging from 915 to 2195 mg/L compared to the DST concentration of 3280 mg/L (Table 1).

For our follow‐up reactor series, we incubated a blend of unfiltered produced fluid and crushed sediment from our field sampling effort at reservoir conditions (47°C and ~1800 psi) with a 15% H_2_/85% CH_4_ gas blend for 1, 3, 7 and 21 days. We incorporated the sediment and extended our reactor timepoints in order to accurately capture immediate and long‐term changes in the fluid geochemistry. For this final series, we used gas chromatography to measure any volatile organic compounds (VOCs) emitted into the headspace as well as completed extensive microbial analysis. We detected a small amount of ethane produced across all the reactors at a low concentration, ranging from 12 to 75 ppm (Table S7). However, no other VOCs were measured. We also used SEM to analyse the sediment from the day 1 and the 21‐day reactors, although no differences were measured between the reactor sediment samples and the field sample sediment.

Microbial loads in the final reactor series were low, ranging from 13 to 801 16S rRNA gene copies/mL of sample (Tables 2 and S2). We were interested in comparing the alpha diversity present in the microbial load of the reactor series with our initial DST fluid sample. We measured this by counting the total number of ASVs and calculating the Shannon richness, Chao1 index and Pielou's evenness (Table 2). For the DST field sample, we report both individual sample replicates as well as pooled samples. Although the pooled sample has more unique total ASVs and a higher Chao1 index than the individual replicates, the Shannon Diversity and Pielou's Evenness are consistent between the pooled sample and the individual replicates. Across all reactor samples, there was an average of 47 unique ASVs per 1046 sequences. Chao1 indices ranged from 18 to 88, Shannon indices ranged from 1.74 to 3.32, and Pielou's evenness measurements ranged from 0.55 to 0.83 per 1046 sequences across all samples. A Wilcoxon rank‐sum test revealed the Shannon Diversity (*p* = 0.001) and Pielou's evenness measurements (*p* = 0.008) were significantly different between the DST fluid samples and the reactor samples, with the reactor samples having lower diversity measurements, although there was no significant difference between the other metrics.

**TABLE 2:** qPCR and alpha diversity metrics for DST fluid and reactor samples. Alpha diversity metrics were calculated after sequence libraries were resampled to the depth of the sample with the fewest sequences from this experiment (1046 sequences).

	16S gene copies/mL	Total ASVs	Chao1 index	Shannon diversity	Pielou's evenness
DST fluid (Pooled replicates)	N/A	124	173	3.72	0.77
DST fluid 200 mL A	4707	73	107	3.40	0.79
DST fluid 200 mL B	10,252	78	89	3.43	0.79
DST fluid 200 mL C	6266	83	99	3.49	0.79
DST fluid 250 mL	260	89	110	3.72	0.83
DST fluid 420 mL	5729	23	23	2.71	0.86
DST fluid 500 mL	211	53	56	3.20	0.81
Reactor Day 1	373	58	66	2.89	0.71
Reactor Day 2	801	61	69	2.51	0.61
Reactor Day 7	495	71	88	2.99	0.70
Reactor Day 21	283	43	43	2.86	0.76
Duplicate reactor Day 1	13	24	24	1.74	0.55
Duplicate reactor Day 2	26	18	18	2.03	0.70
Duplicate reactor Day 7	30	74	78	3.32	0.77
Duplicate reactor Day 21	21	28	28	2.75	0.83

16S rRNA gene amplicon sequencing revealed that the majority of the taxa found in the DST fluid samples were also present in the reactor samples (Figure 3). We identified 36 unique taxa that comprised > 2.5% of one or more samples (Figure 3). Of these taxa, *Porphyrobacter sp*. was by far the most highly prevalent, with an average relative abundance of 26.37% across all samples (Figure 3). *Porphyrobacter sp*. was notably more abundant in reactor samples, with an average of 39.70% across all reactors compared to the field samples average of 10.94%, with replicates running from 4.76% to 14.57%. Although *Porphyrobacter sp*. has been found in subsurface environments that are typically thought of as anaerobic (An et al. 2013; Rollick 2014; Ridley and Voordouw 2018; Ruff et al. 2023), it is classified as an aerobic organism and is often associated with hydrocarbon degradation (Hiraishi and Imhoff 2015). Most other taxa were present at comparable abundances between the DST fluid and reactor samples, with a few exceptions (Figure 3). *Salipaludibacillus sp*. is notable because of its reported high alkaline serine protease production (Ibrahim et al. 2019) and was found in each DST fluid sample replicate with an average relative abundance of 3.70% across all DST fluid samples (Figure 3). However, *Salipaludibacillus sp*. was not present in any of the reactor samples (Figure 3). In contrast, four taxa were present in reactors that were not at detectable levels in any of the DST fluid replicates: *Negativicoccus sp*., env.OPS_17.sp (uncultured Tuber borchii orgin), an Unclassified chitinophagaceae, and *Acinetobacter radioresistens* (Figure 3).

There were several taxa with no or minimal presence in DST fluid and early reactor timepoints, which had comparatively higher relative abundances at later reactor time points. Most notable are *Halomonas sp*., which is a common halophile, and *Micrococcus sp*., which has been reported to degrade environmental pollutants as well as reduce nitrate (Chang and Morris 1962; Dib et al. 2013). *Halomonas sp*. was found at a relative abundance of 0.02% in the pooled DST fluid sample, 0.22% in the Reactor Day 21 sample, 0.33% in the duplicate reactor Day 7 sample, and 5.07% in the duplicate reactor Day 21 sample (Figure 3). *Micrococcus sp*. and an unclassified Dysgonomonadaceae were found at relative abundances of 0.01% and 0.04% in the DST pooled fluid sample (Figure 3). However, *Micrococcus sp*. had a relative abundance of 0.54% in the reactor Day 7 sample and 3.63% in the reactor Day 21 sample, while the unclassified Dysgonomonadaceae sample had a relative abundance of 1.35% in the reactor Day 21 sample and 3.56% in the duplicate reactor Day 2 sample (Figure 3).

We found that each time point from the reactor series and the duplicate reactor series demonstrated the same general geochemical characteristics as the starting reservoir fluid, with two notable exceptions (Table 1). First, the duplicate 21‐day reactor had a comparatively high concentration of iron, at 102.4 mg/L, while all other samples had concentrations below the detection limit (Table 1). This difference may have been due to heterogeneity in the crushed solid. Second, the sulphate concentration present across all the reactor samples was lower than the DST field sample. The DST field sample had a sulphate concentration of 3280 mg/L, while individual reactors had concentrations ranging from 1979 to 2876 mg/L (Table 1). One final notable observation is that it appears there was no or minimal leaching from the core sediment that was included in each reactor, as the overall geochemical compositional trends between the DST field sample and the individual reactor measurements were stable.

To better understand the relationship between the reactor microbiology and geochemistry, we constructed an ordination plot (Figure S3) using NMDS based on Bray–Curtis dissimilarity distances calculated after sequence libraries were subsampled to the lowest sequence depth (1046 sequences). A Mantel test measured no statistically significant correlations between the microbial data and associated geochemistry. However, fitting environmental vectors on our ordination plot revealed that the chloride concentration was significantly correlated (*R* ^2^ = 0.7941, *p* = 0.033) with the microbial data (Figure S3, Table S8).

## 4 Discussion

Research associated with large‐scale hydrogen storage is rapidly growing demand for this commodity and our increasing global need for energy carriers during variable energy production. One of the most promising site types for large‐scale hydrogen storage is deep saline aquifers due to their high storage capacity and recovery ratios (Goodman et al. 2022; Sainz‐Garcia et al. 2017; Al‐Shafi et al. 2023). However, minimal research has been published characterising the changes in geochemistry or microbial communities present in deep saline aquifers when exposed to hydrogen or hydrogen/methane gas blends. Therefore, we collected and characterised samples from a deep saline aquifer from the St. Peter Formation in southern Illinois. In addition to completing a baseline characterisation from the site, we also used an enriched metagenome to identify the presence of genes associated with hydrogen‐consuming reactions as well as completed a 21‐day hydrogen blend short‐term storage simulation study under environmentally relevant conditions.

To understand how subsurface dynamics may change when introduced to hydrogen or hydrogen gas blends, it is essential to characterise the subsurface environment before hydrogen injection occurs. Our SEM and geochemical results revealed that sediment from the core of our deep saline aquifer had high occurrences of sulphur (Figure 1A), while the DST fluid samples had fairly high concentrations of sulphate (3280 mg/L; Table 1). The high availability of sulphur and sulphate in the DST sediment and fluid (Figure 1A,B, Table 1) suggests potential for sulphate reduction during hydrogen‐storage conditions. However, common sulphur and sulphate reducing taxa including *Desulfomonas, Desulfobacter, Desulfohalobium, Halanaerobium, Orenia* and *Marinobacter* (Dopffel et al. 2021; Booker et al. 2017; Tinker et al. 2020) were not present in the 16S rRNA sequences from our field sample (Figure 3). Therefore, we opted to analyse the metagenome of an enriched field sample to identify the presence of sulphate reducing genes as well as genes associated with common hydrogen‐consuming reactions.

Methanogenesis and acetogenesis are the most likely hydrogen‐consuming reactions to occur in underground hydrogen storage environments (Goodman et al. 2022; Dopffel et al. 2021; Ebigbo et al. 2013; McGenity 2010; Tyne et al. 2021; Aftab et al. 2022; Jafari Raad et al. 2022). No genes associated with methanogenesis were present, and genes associated with acetogenesis via the Wood‐Ljungdahl pathway were either absent or less than 50% complete for all MAGs (Figure 2). This suggests methanogenesis is not a major concern at the Lively Grove site, although acetogenesis may occur under certain conditions. In addition to consuming hydrogen, sulphate reduction is associated with many infrastructure and operational hazards, including microbial‐induced corrosion (MIC) (Liang et al. 2014; Loto 2017; Kotu et al. 2019; Xu et al. 2023), H_2_S production and FeS precipitation (Dopffel et al. 2021; Aftab et al. 2022; Jafari Raad et al. 2022), with secondary reactions from these events impacting well permeability (Zivar et al. 2021; Dopffel et al. 2021; Aftab et al. 2022; Jafari Raad et al. 2022). However, our metagenomic analysis suggests these activities are less likely to occur at our field site as there is no complete sulphur reduction pathway present across all 9 MAGs. To complete our metagenomic analysis, we also examined our MAGs for denitrification and iron reduction genes. Although they are less likely to occur than the other reactions, denitrification and iron reduction are hydrogen‐consuming reactions that can cause corrosion and other operational hazards (Xu et al. 2023; Kielemoes et al. 2000). However, our MAGs had an absence of iron reduction genes and minimal denitrification genes, suggesting this activity is unlikely at our field site during hydrogen storage scenarios.

Beyond understanding the metabolic potential for hydrogen‐consuming reactions through an enriched field sample, we were also interested in completing a reactor series in order to capture geochemical, microbiological and gas measurements at various time points throughout the duration of storage. This would allow us to decouple abiotic and biotic processes as well as allow us to confirm the results of the metagenomic analysis of our enriched field sample, which suggested that common hydrogen‐reducing reactions would either not occur (methanogenesis and iron reduction) or be unlikely to rapidly occur (acetogenesis, sulphate reduction and denitrification) during hydrogen storage conditions. This was particularly important as the enriched field sample is only an approximation of the in situ microbial community. Additionally, metagenomics captures the functional potential of a sample, but it does not capture how a microbial community may change in both composition and biomass over time under varying conditions or how the expression of particular genes may be altered, especially in pathways that are partially complete in individual MAGs, but complete across the entire microbial community.

Methanogenesis and acetogenesis are most likely to occur in environments with high temperatures, a lower pH (< 7), and high hydrogen concentrations (Dopffel et al. 2021; Aftab et al. 2022; Wilhelms et al. 2001). The pH of the DST reservoir fluid was 7.3, and our reactor experiments used a gas blend with a fairly low partial pressure of hydrogen (15% H_2_/85% CH_4_ gas blend) at a moderate temperature (47°C). Thus, our reactor conditions, which modelled the field site conditions, were not primed to select for a high occurrence of methanogenesis or acetogenesis. Consistent with this, our reactor samples had a low biomass (13 to 801 16S rRNA gene copies/mL of sample) throughout the 21‐day experiment. This suggests that during short‐term storage, high‐withdrawal scenarios, hydrogen is not a powerful enough electron donor in this environment to see an increase in microbial biomass. No archaea were detected in any of our field samples or reactor samples using the universal V4 515F/806R primers, suggesting our initial site and reactor experiments did not have a high metabolic capacity to complete methanogenesis. Only one known acetogen, *Acetobacterium sp*. was found in some of the DST fluid samples and was only present in half of the reactors and at lower abundances than the DST fluid samples (Figure 3), suggesting that acetogenesis was not a primary reaction occurring in our short‐term reactor study. Additionally, in our final reactor series, the only VOC measured was ethane, suggesting no or minimal fermentation during short‐term hydrogen storage scenarios.

One notable aspect of the reactor geochemical data is that the sulphate values were lower than the initial field sample. We also measured a loss of 2.5% to 4.37% hydrogen gas across all reactors. This initially suggested rapid sulphate reduction, with hydrogen sulphide as a likely product. However, the loss of hydrogen was observed across both abiotic and biotic reactors, suggesting the observed hydrogen loss is an abiotic sink, rather than microbially driven consumption. This was supported by (1) a lack of common sulphate reducers in the 16S rRNA sequences obtained from the reactor series, (2) a microbial community that remained consistent throughout the reactor series and (3) no significant correlations between the sulphate geochemistry and the microbial data. Upon further investigation, we found that the lowered sulphate measurements were highly variable and exhibited no clear trends across the reactor treatments. This suggests that a ripe area for future work is refining existing methods of raw fluid preservation, especially as it pertains to use in reactor studies. During the field sample collection process, we utilised previously published methods (Tinker et al. 2022, 2020; Lipus et al. 2017, 2018, 2019) to capture and preserve samples in order to reduce bottle effects. However, produced fluid is a complex brine solution and it is possible there was separation of the sulphate due to fluid heterogeneity while aliquoting the fluid into the different storage bottles in the field. This could have been further impacted by precipitation, complexation, and/or sorption, which are difficult to understand. A further possibility is that the variable storage times of the aliquoted raw fluid impacted the sulphate measurements. Overall, this demonstrates that sulphate measurements in this system are unusually sensitive and that future work should consider methods to test the variability in these measurements.

Our goal was to identify potential geochemical and microbial impacts of hydrogen storage in deep saline aquifers through the characterisation of field samples and complementary enrichment and reactor experiments. Given the high biological reactivity of hydrogen, this first study was to determine if short‐term storage would be impacted by the natural microbial community in the storage reservoir. We detected no sulphur or sulphate‐reducing taxa and few microorganisms known to be hydrogen‐consumers, demonstrating how challenging it will be to understand the potential microbial interactions in this system. Our data suggest that short‐term hydrogen storage at this field site poses minimal risk of rapid microbial hydrogen‐consumption, further suggesting that temporary storage during high usage seasons may occur with minimal microbial consumption. However, future work is required to determine if this minimal risk to short‐term storage can be expected in other subsurface storage reservoirs. Prior work in subsurface environments demonstrate that the microbial community is highly dynamic and can cause major impacts, if conditions are altered or if it is allowed to adapt over extended periods of time. Therefore, it is important to note that long‐term hydrogen storage expected to occur during low usage seasons may result in hydrogen‐reduction through common microbial hydrogen‐consumption reactions, as they are highly energetically favourable. Overall, our work highlights the challenges that await us as we begin to develop and use large‐scale hydrogen storage facilities.

## Author Contributions

**Kara A. Tinker:** conceptualization, investigation, writing – original draft, methodology, visualization, writing – review and editing, data curation. **Winston Anthony:** data curation, methodology, writing – review and editing. **Meghan Brandi:** methodology, visualization, writing – review and editing, data curation. **Sam Flett:** methodology, visualization, writing – review and editing, data curation. **Christopher E. Bagwell:** writing – review and editing. **Chuck Smallwood:** writing – review and editing. **Ryan Davis:** writing – review and editing. **Djuna Gulliver:** conceptualization, investigation, funding acquisition, writing – review and editing, methodology.

## Conflicts of Interest

The authors declare no conflicts of interest.

## Supporting information


**Data S1.** Supporting Information.

## Data Availability

The 16S rRNA gene amplicon sequences and metagenome sequences generated from this experiment were submitted to the NCBI Sequence Read Archive and are available under accession number PRJNA962587.
