# Supplementary material for: Identifying Potential Geochemical and Microbial Impacts of Hydrogen Storage in a Deep Saline Aquifer
Source: Environ Microbiol Rep. 2025 Apr 15;17(2):e70076. doi: 10.1111/1758-2229.70076 (PMC11999705; doi:10.1111/1758-2229.70076)
Supplement: Supplementary file 1 — Data S1. Supporting Information. [file EMI4-17-e70076-s001.pdf]

SI Data 1. gBlocks Gene Fragment Sequence for qPCR Standard. Fragment was constructed using the 16S bac, McrA, DsrA, and RdlA genes.

ACAAAGAAGCCCACAGCCGACTTGGTTTCTACGGTTACGACCTGCAGGACCAGTGCGGAGCATCCAACTCTCTCTC
CATCAGGAGTGACGAAGGTCTGATCCACGAATTACGTGGTCCTAACTACCCTAACTACGCCATGAAAAACGCTGGT
CTCTCCGCATGGTATCTCTGTATGTACCTGCACAAGGAAGGTCACGGACGTCTGGGATTCTTCGGATTCGACTTGC
AGGACCAGTGTGGTGCAACCAACACCTTCTCCTACCAATCCGACGAAGGTCTTGATGACTTGACGTCATCCCCACC
TTCCTCCCCGTTAACCGGGGCGGTCTCCATAGAGTGCCCAGCATTACCTGGTAGCAACTATGAACAGGGGTTGCGC
TCGTTGCGGGACTTAACCCAACACCTCACGGCACGAGCTGACGACAGCCATGCAGCACCTGTCTCGGGGGCGTCA
TCGGCCGTTACTGTGACCAGCCCGAACAGTTCCCCGGCGTGGCGCACTTCCACACCGTGCGCGTGAACCAGCCCG
CGGCGAAGTACTACCACACCGACTACCTGCGCCAGCTCTGCGACCTGTGGGACCTGCGCGGCTCCGGTCTGACCA
ACATGCACGGTTCCACCGGCGACATCGAGTATTTTGGACAGCTGCCTCAGGATAAGAAGATAGTTGTTTACTGCTA
TACAGGTCAAACAGCAGGTCAGGCAGTAGCAGGTTTAAGAATGTTAGACTATGATGCTGTATCACTTAATGGTGG
TATGGGTACTTCTGCTAATGAGCCT

SI Table 1. Quality Control information for 16S sequencing Data. Samples ultimately removed from the dataset due to poor quality or low depth are shown in red.

File Name	Miseq Run	Input	Filtered	% of input passed filter	denoised	non-chimeric	percentage of input non-chimeric	sequences after filtering mitochondria & chloroplast	Pruned in R
Dec_BA_D1_rep1	Dec_2022	12157	4633	38.11	4597	4597	37.81	4597	N
Dec_BA_D1_rep2	Dec_2022	14045	3518	25.05	3488	3488	24.83	3488	N
Dec_BA_D2_rep1	Dec_2022	22203	4388	19.76	4292	4292	19.33	4292	N
Dec_BA_D2_rep2	Dec_2022	10891	5380	49.4	5341	5341	49.04	5341	N
Dec_BA_D21_rep1	Dec_2022	11121	4059	36.5	4013	3993	35.91	3993	N
Dec_BA_D21_rep2	Dec_2022	22831	3102	13.59	3051	3021	13.23	3021	N
Dec_BA_D21_rep3	Dec_2022	17444	1284	7.36	1267	1260	7.22	1260	N
Dec_BA_D7_rep1	Dec_2022	31693	5356	16.9	5282	5260	16.6	5222	N
Dec_BA_D7_rep2	Dec_2022	6163	2345	38.05	2312	2312	37.51	2312	N
Dec_BB_D2_rep2	Dec_2022	8646	2884	33.36	2866	2866	33.15	2866	N
Dec_BB_D21_rep2	Dec_2022	56025	1129	2.02	1059	1059	1.89	1046	N
Dec_BB_D7_rep2	Dec_2022	239227	5997	2.51	5920	5843	2.44	5779	N
Dec_BB_D7_rep3	Dec_2022	343906	2325	0.68	2224	2224	0.65	2224	N
Dec_DST_Fluid_200mL_A	Dec_2022	15610	6900	44.2	6797	6721	43.06	6721	N
Dec_DST_Fluid_200mL_B	Dec_2022	14134	6524	46.16	6386	6386	45.18	6386	N
Dec_DST_Fluid_200mL_C	Dec_2022	17722	5235	29.54	5120	5120	28.89	5104	N
Dec_DST_Fluid_250mL	Dec_2022	11925	1746	14.64	1706	1706	14.31	1659	N
Dec_DST_Fluid_420mL	Dec_2022	18511	8413	45.45	8305	8305	44.87	8280	N
Dec_DST_Fluid_500mL	Dec_2022	23825	4435	18.61	4340	3953	16.59	3464	N
March_BA_D1	March_2022	14965	11731	78.39	11638	11638	77.77	11638	N
March_BA_D2	March_2022	15911	12485	78.47	12396	12396	77.91	12396	N
March_BA_D7	March_2022	20068	17130	85.36	17065	17065	85.04	17065	N
March_BB_D1	March_2022	20707	15433	74.53	15360	15360	74.18	15360	N
March_DST_Fluid_200mL_A	March_2022	16494	14456	87.64	14414	14414	87.39	14414	N
March_DST_Fluid_200mL_B	March_2022	13713	10321	75.26	10265	10086	73.55	10086	N
March_DST_Fluid_200mL_C	March_2022	13488	9986	74.04	9915	9235	68.47	9235	N
March_DST_Fluid_500mL	March_2022	10210	7200	70.52	7142	7142	69.95	7142	N
Dec_BB_D1_rep1	Dec_2022	25601	878	3.43	815	815	3.18	815	Y
Dec_BB_D21_rep1	Dec_2022	125848	3004	2.39	2905	2847	2.26	2847	Y
Dec_BB_D21_rep3	Dec_2022	43295	528	1.22	445	445	1.03	445	Y
Dec_Combined_Kit_Blank_4_PCR_Blank_2	Dec_2022	10442	2114	20.25	2078	2078	19.9	2078	Y
Dec_Kit_Blank_2	Dec_2022	17240	3311	19.21	3278	3278	19.01	3267	Y
Dec_Kit_Blank_3	Dec_2022	20697	3583	17.31	3518	3518	17	3505	Y
Dec_Kit_Blank_4	Dec_2022	21322	4476	20.99	4405	4398	20.63	4398	Y
Dec_PCR_Blank_1	Dec_2022	11378	3909	34.36	3897	3897	34.25	3665	Y
Dec_PCR_Blank_3_rep1	Dec_2022	22744	4205	18.49	4106	4106	18.05	3996	Y
Dec_PCR_Blank_3_rep2	Dec_2022	83274	562	0.67	487	487	0.58	487	Y
March_BA_D21	March_2022	81	12	14.81	2	2	2.47	2	Y
March_BB_D2	March_2022	23	2	8.7	2	2	8.7	2	Y
March_BB_D21	March_2022	13	0	0	0	0	0	#N/A	Y
March_BB_D7	March_2022	16	0	0	0	0	0	#N/A	Y
March_DST_Fluid_250mL	March_2022	6	0	0	0	0	0	#N/A	Y
March_DST_Fluid_420mL	March_2022	10839	9854	90.91	9796	8178	75.45	8178	N
March_Kit_Blank_1	March_2022	3788	1579	41.68	1565	1565	41.31	1565	Y
March_Kit_Blank_1	March_2022	3635	3337	91.8	3329	2975	81.84	2975	Y
March_Kit_Blank_2	March_2022	373	239	64.08	233	233	62.47	233	Y
March_Kit_Blank_3	March_2022	336	247	73.51	241	241	71.73	241	Y
March_Kit_Blank_4	March_2022	5874	5083	86.53	5066	5066	86.24	5066	Y
March_Kit_Blank_5	March_2022	23309	18595	79.78	18553	15741	67.53	15741	Y
March_PCR_Blank_1	March_2022	248291	169148	68.12	168458	151711	61.1	151669	Y
March_PCR_Blank_2	March_2022	565	176	31.15	163	163	28.85	163	Y
March_PCR_Blank_3	March_2022	147	67	45.58	62	62	42.18	62	Y
March_PCR_Blank_4	March_2022	318	222	69.81	219	219	68.87	219	Y
March_PCR_Blank_5	March_2022	1272	938	73.74	931	931	73.19	931	Y
March_PCR_Blank_6	March_2022	4658	2636	56.59	2552	2476	53.16	2476	Y

Table 2. qPCR and quality contorl information for 16S sequencing

Sample Name	16S gene copies/mL sample	Input	Filtered	denoised	non-chimeric	sequences after filtering mitochondria & chloroplast
DST Fluid 200 mL A	4707	32104	21356	21211	21135	21135
DST Fluid 200 mL B	10252	27847	16845	16651	16472	16472
DST Fluid 200 mL C	6266	31210	15221	15035	14355	14339
DST Fluid 250 mL	260	11925	1746	1706	1706	1659
DST Fluid 420 mL	5729	18511	8413	8305	8305	8280
DST Fluid 500 mL	211	34035	11635	11482	11095	10606
Reactor Day 1	373	41167	19882	19723	19723	19723
Reactor Day 2	801	49005	22253	22029	22029	22029
Reactor Day 7	495	57924	24831	24659	24637	24599
Reactor Day 21	283	51396	8445	8331	8274	8274
Duplicate Reactor Day 1	13	20707	15433	15360	15360	15360
Duplicate Reactor Day 2	26	8646	2884	2866	2866	2866
Duplicate Reactor Day 7	30	583133	8322	8144	8067	8003
Duplicate Reactor Day 21	21	56025	1129	1059	1059	1046

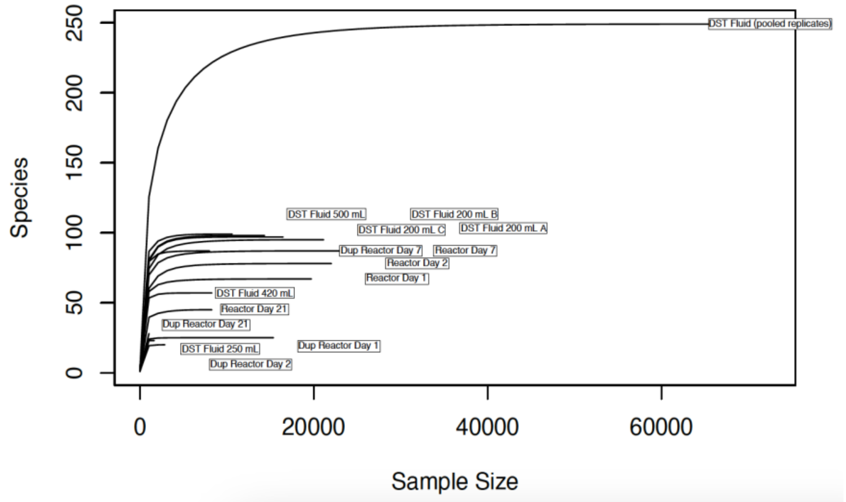

SI Figure 1. Rarefaction curve for samples at a sequencing depth of 1046 sequences per sample

SI Table 3. Rarefaction curve statistics for samples.

Sample Name	Number of species at a depth of 1047 sequences	Number of species at maximum depth
DST Fluid (Pooled Replicates)	126	249
DST Fluid 200 mL A	74	95
DST Fluid 200 mL B	80	97
DST Fluid 200 mL C	80	98
DST Fluid 250 mL	87	99
DST Fluid 420 mL	23	23
DST Fluid 500 mL	54	57
Reactor Day 1	58	67
Reactor Day 2	60	78
Reactor Day 7	70	87
Reactor Day 21	40	45
Duplicate Reactor Day 1	24	25
Duplicate Reactor Day 2	19	20
Duplicate Reactor Day 7	80	87
Duplicate Reactor Day 21	28	28

SI Table 4. Quality information for the recovered MAGs.

Lowest Classification	completeness (%)	contamination (%)	GC	N50	Size (bp)	Closest Placement ANI (%)	Closest placement taxa
Acinetobacter sp.	55.25	1.64	0.406	2062	2066424	N/A	N/A
Erythrobacter sp001724215	99.31	0.458	0.619	88614	3076757	97.4	d__Bacteria;p__Proteobacteria;c__Alphaproteobacteria;o__Sphingomonadales;f__Sphingomonadaceae;g__Erythrobacter;s__Erythrobacter sp001724215
Herbaspirillum huttiense	99.87	0.056	0.622	418313	6130777	97.51	d__Bacteria;p__Proteobacteria;c__Gammaproteobacteria;o__Burkholderiales;f__Burkholderiaceae;g__Herbaspirillum;s__Herbaspirillum huttiense
Lactococcus lactis E	76.71	1.163	0.354	15352	1860159	97.94	d__Bacteria;p__Firmicutes;c__Bacilli;o__Lactobacillales;f__Streptococcaceae;g__Lactococcus;s__Lactococcus lactis_E
Microcella alkaliphila A	63.79	0.084	0.683	3931	1544139	97.53	d__Bacteria;p__Actinobacteriota;c__Actinomycetia;o__Actinomycetales;f__Microbacteriaceae;g__Microcella;s__Microcella alkaliphila_A
Paracoccus sp.	95.83	0.757	0.671	24377	3303234	83.91	d__Bacteria;p__Proteobacteria;c__Alphaproteobacteria;o__Rhodobacterales;f__Rhodobacteraceae;g__Paracoccus;s__Paracoccus tibetensis
Sphingomonas sp003355005	92.65	2.104	0.676	11448	3350576	99.94	d__Bacteria;p__Proteobacteria;c__Alphaproteobacteria;o__Sphingomonadales;f__Sphingomonadaceae;g__Sphingomonas;s__Sphingomonas sp003355005
Sphingomonas sp005503355	84.29	2.847	0.669	5168	3903690	99.92	d__Bacteria;p__Proteobacteria;c__Alphaproteobacteria;o__Sphingomonadales;f__Sphingomonadaceae;g__Sphingomonas;s__Sphingomonas sp005503355
Streptococcus thermophilus	92.73	1.291	0.392	33984	1604276	98.57	d__Bacteria;p__Firmicutes;c__Bacilli;o__Lactobacillales;f__Streptococcaceae;g__Streptococcus;s__Streptococcus thermophilus

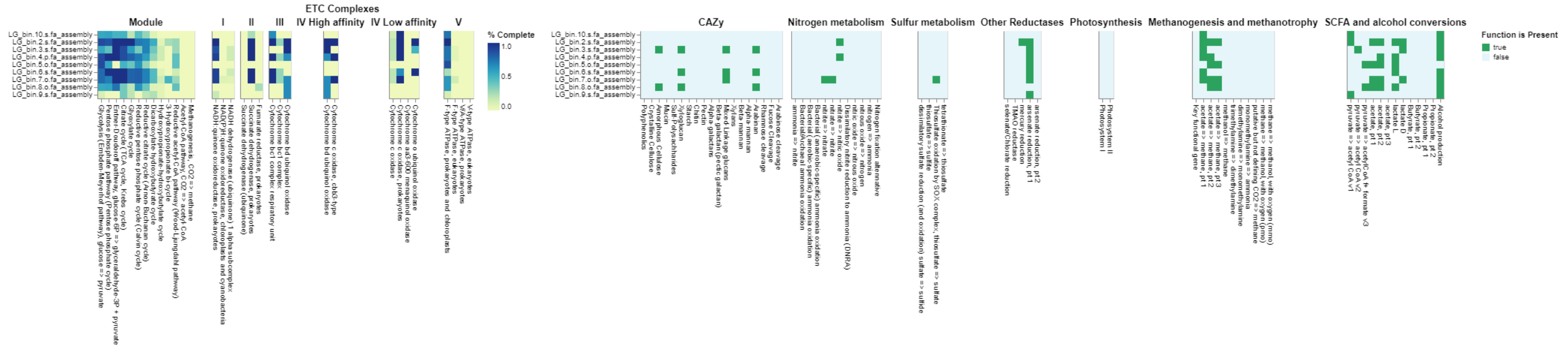

SI Figure 2. Full DRAM output for the MAGs obtained from the enriched field sample.

SI Table 5. Complete table showing the KO identifier, gene description, and presence/absence information for genes associated with methanogenesis, acetogenesis, sulfate reduction, denitrification, and iron transportation. Note that only genes with a presence in at least one MAG are displayed.

	Gene ID	Gene Description	Module	Header	*Acinetobacter sp.*	*Erythrobacter sp001724215*	*Herbaspirillum huttiense*	*Lactococcus lactis E*	*Microcella alkaliphila A*	*Paracoccus sp.*	*Sphingomonas sp003355005*	*Sphingomonas sp005503355*	*Streptococcus thermophilus*
Methanogenesis	K00125	formate dehydrogenase (coenzyme F420) [EC:1.8.98.6] [RN:R11944]	Methanogenesis, CO2 => methane	C1-methane	0	0	0	0	0	0	0	0	0
	K00200	formylmethanofuran dehydrogenase [EC:1.2.99.5] [RN:R03015]	Methanogenesis, CO2 => methane	C1-methane	0	0	0	0	0	0	0	0	0
	K00201	formylmethanofuran dehydrogenase [EC:1.2.99.5] [RN:R03015]	Methanogenesis, CO2 => methane	C1-methane	0	0	0	0	0	0	0	0	0
	K00202	formylmethanofuran dehydrogenase [EC:1.2.99.5] [RN:R03015]	Methanogenesis, CO2 => methane	C1-methane	0	0	0	0	0	0	0	0	0
	K00203	formylmethanofuran dehydrogenase [EC:1.2.99.5] [RN:R03015]	Methanogenesis, CO2 => methane	C1-methane	0	0	0	0	0	0	0	0	0
	K00204	4Fe-4S ferredoxin	Methanogenesis, CO2 => methane	C1-methane	0	0	0	0	0	0	0	0	0
	K00205	4Fe-4S ferredoxin	Methanogenesis, CO2 => methane	C1-methane	0	0	0	0	0	0	0	0	0
	K00319	methylenetetrahydromethanopterin dehydrogenase [EC:1.5.99.9] [RN:R04456]	Methanogenesis, CO2 => methane	C1-methane	0	0	0	0	0	0	0	0	0
	K00320	coenzyme F420-dependent N5,N10-methenyltetrahydromethanopterin reductase [EC:1.5.99.11] [RN:R04464]	Methanogenesis, CO2 => methane	C1-methane	0	0	0	0	0	0	0	0	0
	K00399	methyl-coenzyme M reductase [EC:2.8.4.1] [RN:R04541]	Methanogenesis, CO2 => methane	C1-methane	0	0	0	0	0	0	0	0	0
	K00401	methyl-coenzyme M reductase [EC:2.8.4.1] [RN:R04541]	Methanogenesis, CO2 => methane	C1-methane	0	0	0	0	0	0	0	0	0
	K00402	methyl-coenzyme M reductase [EC:2.8.4.1] [RN:R04541]	Methanogenesis, CO2 => methane	C1-methane	0	0	0	0	0	0	0	0	0
	K00577	tetrahydromethanopterin S-methyltransferase [EC:2.1.1.86] [RN:R04347]	Methanogenesis, CO2 => methane	C1-methane	0	0	0	0	0	0	0	0	0
	K00578	tetrahydromethanopterin S-methyltransferase [EC:2.1.1.86] [RN:R04347]	Methanogenesis, CO2 => methane	C1-methane	0	0	0	0	0	0	0	0	0
	K00579	tetrahydromethanopterin S-methyltransferase [EC:2.1.1.86] [RN:R04347]	Methanogenesis, CO2 => methane	C1-methane	0	0	0	0	0	0	0	0	0
	K00580	tetrahydromethanopterin S-methyltransferase [EC:2.1.1.86] [RN:R04347]	Methanogenesis, CO2 => methane	C1-methane	0	0	0	0	0	0	0	0	0
	K00581	tetrahydromethanopterin S-methyltransferase [EC:2.1.1.86] [RN:R04347]	Methanogenesis, CO2 => methane	C1-methane	0	0	0	0	0	0	0	0	0
	K00582	tetrahydromethanopterin S-methyltransferase [EC:2.1.1.86] [RN:R04347]	Methanogenesis, CO2 => methane	C1-methane	0	0	0	0	0	0	0	0	0
	K00583	tetrahydromethanopterin S-methyltransferase [EC:2.1.1.86] [RN:R04347]	Methanogenesis, CO2 => methane	C1-methane	0	0	0	0	0	0	0	0	0
	K00584	tetrahydromethanopterin S-methyltransferase [EC:2.1.1.86] [RN:R04347]	Methanogenesis, CO2 => methane	C1-methane	0	0	0	0	0	0	0	0	0
	K00672	formylmethanofuran--tetrahydromethanopterin N-formyltransferase [EC:2.3.1.101] [RN:R03390]	Methanogenesis, CO2 => methane	C1-methane	0	0	0	0	0	0	0	0	0
	K01499	methenyltetrahydromethanopterin cyclohydrolase [EC:3.5.4.27] [RN:R03464]	Methanogenesis, CO2 => methane	C1-methane	0	0	0	0	0	0	0	0	0
	K03388	heterodisulfide reductase 2 [EC:1.8.7.3 1.8.98.4 1.8.98.5 1.8.98.6] [RN:R11928 R11931 R11943 R11944]	Methanogenesis, CO2 => methane	C1-methane	0	0	0	0	0	0	0	0	0
	K03389	heterodisulfide reductase 2 [EC:1.8.7.3 1.8.98.4 1.8.98.5 1.8.98.6] [RN:R11928 R11931 R11943 R11944]	Methanogenesis, CO2 => methane	C1-methane	0	0	0	0	0	0	0	0	0
	K03390	heterodisulfide reductase 2 [EC:1.8.7.3 1.8.98.4 1.8.98.5 1.8.98.6] [RN:R11928 R11931 R11943 R11944]	Methanogenesis, CO2 => methane	C1-methane	0	0	0	0	0	0	0	0	0
	K08264	heterodisulfide reductase [EC:1.8.98.1] [RN:R04540]	Methanogenesis, CO2 => methane	C1-methane	0	0	0	0	0	0	0	0	0
	K08265	heterodisulfide reductase [EC:1.8.98.1] [RN:R04540]	Methanogenesis, CO2 => methane	C1-methane	0	0	0	0	0	0	0	0	0
	K11260	4Fe-4S ferredoxin	Methanogenesis, CO2 => methane	C1-methane	0	0	0	0	0	0	0	0	0
	K11261	formylmethanofuran dehydrogenase [EC:1.2.99.5] [RN:R03015]	Methanogenesis, CO2 => methane	C1-methane	0	0	0	0	0	0	0	0	0
	K13942	5,10-methenyltetrahydromethanopterin hydrogenase [EC:1.12.98.2] [RN:R04455]	Methanogenesis, CO2 => methane	C1-methane	0	0	0	0	0	0	0	0	0
	K14126	F420-non-reducing hydrogenase [EC:1.8.98.5] [RN:R11943]	Methanogenesis, CO2 => methane	C1-methane	0	0	0	0	0	0	0	0	0
	K14127	F420-non-reducing hydrogenase iron-sulfur subunit [EC:1.8.98.5 1.8.98.6] [RN:R11943 R11944]	Methanogenesis, CO2 => methane	C1-methane	0	0	0	0	0	0	0	0	0
	K14128	F420-non-reducing hydrogenase [EC:1.8.98.5] [RN:R11943]	Methanogenesis, CO2 => methane	C1-methane	0	0	0	0	0	0	0	0	0
	K22480	heterodisulfide reductase 1 [EC:1.8.7.3] [RN:R11931]	Methanogenesis, CO2 => methane	C1-methane	0	0	0	0	0	0	0	0	0
	K22481	heterodisulfide reductase 1 [EC:1.8.7.3] [RN:R11931]	Methanogenesis, CO2 => methane	C1-methane	0	0	0	0	0	0	0	0	0
	K22482	heterodisulfide reductase 1 [EC:1.8.7.3] [RN:R11931]	Methanogenesis, CO2 => methane	C1-methane	0	0	0	0	0	0	0	0	0
	K22516	formate dehydrogenase (coenzyme F420) [EC:1.8.98.6] [RN:R11944]	Methanogenesis, CO2 => methane	C1-methane	0	0	0	0	0	0	0	0	0
	K00125	formate dehydrogenase (coenzyme F420) [EC:1.8.98.6] [RN:R11944]	Methanogenesis, acetate => methane	C1-methane	0	0	0	0	0	0	0	0	0
	K00193	acetyl-CoA decarbonylase/synthase complex [EC:2.3.1.-] [RN:R09096]	Methanogenesis, acetate => methane	C1-methane	0	0	0	0	0	0	0	0	0
	K00194	acetyl-CoA decarbonylase/synthase complex [EC:2.3.1.-] [RN:R09096]	Methanogenesis, acetate => methane	C1-methane	0	0	0	0	0	0	0	0	0
	K00197	acetyl-CoA decarbonylase/synthase complex [EC:2.3.1.-] [RN:R09096]	Methanogenesis, acetate => methane	C1-methane	0	0	0	0	0	0	0	0	0
	K00399	methyl-coenzyme M reductase [EC:2.8.4.1] [RN:R04541]	Methanogenesis, acetate => methane	C1-methane	0	0	0	0	0	0	0	0	0
	K00401	methyl-coenzyme M reductase [EC:2.8.4.1] [RN:R04541]	Methanogenesis, acetate => methane	C1-methane	0	0	0	0	0	0	0	0	0
	K00402	methyl-coenzyme M reductase [EC:2.8.4.1] [RN:R04541]	Methanogenesis, acetate => methane	C1-methane	0	0	0	0	0	0	0	0	0
	K00577	tetrahydromethanopterin S-methyltransferase [EC:2.1.1.86] [RN:R04347]	Methanogenesis, acetate => methane	C1-methane	0	0	0	0	0	0	0	0	0
	K00578	tetrahydromethanopterin S-methyltransferase [EC:2.1.1.86] [RN:R04347]	Methanogenesis, acetate => methane	C1-methane	0	0	0	0	0	0	0	0	0
	K00579	tetrahydromethanopterin S-methyltransferase [EC:2.1.1.86] [RN:R04347]	Methanogenesis, acetate => methane	C1-methane	0	0	0	0	0	0	0	0	0
	K00580	tetrahydromethanopterin S-methyltransferase [EC:2.1.1.86] [RN:R04347]	Methanogenesis, acetate => methane	C1-methane	0	0	0	0	0	0	0	0	0
	K00581	tetrahydromethanopterin S-methyltransferase [EC:2.1.1.86] [RN:R04347]	Methanogenesis, acetate => methane	C1-methane	0	0	0	0	0	0	0	0	0
	K00582	tetrahydromethanopterin S-methyltransferase [EC:2.1.1.86] [RN:R04347]	Methanogenesis, acetate => methane	C1-methane	0	0	0	0	0	0	0	0	0
	K00583	tetrahydromethanopterin S-methyltransferase [EC:2.1.1.86] [RN:R04347]	Methanogenesis, acetate => methane	C1-methane	0	0	0	0	0	0	0	0	0
	K00584	tetrahydromethanopterin S-methyltransferase [EC:2.1.1.86] [RN:R04347]	Methanogenesis, acetate => methane	C1-methane	0	0	0	0	0	0	0	0	0
	K00625	phosphate acetyltransferase [EC:2.3.1.8] [RN:R00230]	Methanogenesis, acetate => methane	C1-methane	0	2	1	1	0	0	0	0	1
	K00925	acetate kinase [EC:2.7.2.1] [RN:R00315]	Methanogenesis, acetate => methane	C1-methane	0	1	1	2	0	1	0	0	1
	K01895	acetyl-CoA synthetase [EC:6.2.1.1] [RN:R00235]	Methanogenesis, acetate => methane	C1-methane	0	1	2	0	1	1	1	1	0
	K03388	heterodisulfide reductase 2 [EC:1.8.7.3 1.8.98.4 1.8.98.5 1.8.98.6] [RN:R11928 R11931 R11943 R11944]	Methanogenesis, acetate => methane	C1-methane	0	0	0	0	0	0	0	0	0
	K03389	heterodisulfide reductase 2 [EC:1.8.7.3 1.8.98.4 1.8.98.5 1.8.98.6] [RN:R11928 R11931 R11943 R11944]	Methanogenesis, acetate => methane	C1-methane	0	0	0	0	0	0	0	0	0
	K03390	heterodisulfide reductase 2 [EC:1.8.7.3 1.8.98.4 1.8.98.5 1.8.98.6] [RN:R11928 R11931 R11943 R11944]	Methanogenesis, acetate => methane	C1-methane	0	0	0	0	0	0	0	0	0
	K08264	heterodisulfide reductase [EC:1.8.98.1] [RN:R04540]	Methanogenesis, acetate => methane	C1-methane	0	0	0	0	0	0	0	0	0
	K08265	heterodisulfide reductase [EC:1.8.98.1] [RN:R04540]	Methanogenesis, acetate => methane	C1-methane	0	0	0	0	0	0	0	0	0
	K13788	phosphate acetyltransferase [EC:2.3.1.8] [RN:R00230]	Methanogenesis, acetate => methane	C1-methane	0	0	0	0	0	0	0	0	0
	K14126	F420-non-reducing hydrogenase [EC:1.8.98.5] [RN:R11943]	Methanogenesis, acetate => methane	C1-methane	0	0	0	0	0	0	0	0	0
	K14127	F420-non-reducing hydrogenase iron-sulfur subunit [EC:1.8.98.5 1.8.98.6] [RN:R11943 R11944]	Methanogenesis, acetate => methane	C1-methane	0	0	0	0	0	0	0	0	0
	K14128	F420-non-reducing hydrogenase [EC:1.8.98.5] [RN:R11943]	Methanogenesis, acetate => methane	C1-methane	0	0	0	0	0	0	0	0	0
	K22480	heterodisulfide reductase 1 [EC:1.8.7.3] [RN:R11931]	Methanogenesis, acetate => methane	C1-methane	0	0	0	0	0	0	0	0	0
	K22481	heterodisulfide reductase 1 [EC:1.8.7.3] [RN:R11931]	Methanogenesis, acetate => methane	C1-methane	0	0	0	0	0	0	0	0	0
	K22482	heterodisulfide reductase 1 [EC:1.8.7.3] [RN:R11931]	Methanogenesis, acetate => methane	C1-methane	0	0	0	0	0	0	0	0	0
	K22516	formate dehydrogenase (coenzyme F420) [EC:1.8.98.6] [RN:R11944]	Methanogenesis, acetate => methane	C1-methane	0	0	0	0	0	0	0	0	0
	K16954	mtsA; methylthiol:coenzyme M methyltransferase [EC:2.1.1.251]	Methanogenesis, methanethiol => methane	C1-methane	0	0	0	0	0	0	0	0	0
	K00125	formate dehydrogenase (coenzyme F420) [EC:1.8.98.6] [RN:R11944]	Methanogenesis, methanol => methane	C1-methane	0	0	0	0	0	0	0	0	0
	K00399	methyl-coenzyme M reductase [EC:2.8.4.1] [RN:R04541]	Methanogenesis, methanol => methane	C1-methane	0	0	0	0	0	0	0	0	0
	K00401	methyl-coenzyme M reductase [EC:2.8.4.1] [RN:R04541]	Methanogenesis, methanol => methane	C1-methane	0	0	0	0	0	0	0	0	0
	K00402	methyl-coenzyme M reductase [EC:2.8.4.1] [RN:R04541]	Methanogenesis, methanol => methane	C1-methane	0	0	0	0	0	0	0	0	0
	K03388	heterodisulfide reductase 2 [EC:1.8.7.3 1.8.98.4 1.8.98.5 1.8.98.6] [RN:R11928 R11931 R11943 R11944]	Methanogenesis, methanol => methane	C1-methane	0	0	0	0	0	0	0	0	0
	K03389	heterodisulfide reductase 2 [EC:1.8.7.3 1.8.98.4 1.8.98.5 1.8.98.6] [RN:R11928 R11931 R11943 R11944]	Methanogenesis, methanol => methane	C1-methane	0	0	0	0	0	0	0	0	0
	K03390	heterodisulfide reductase 2 [EC:1.8.7.3 1.8.98.4 1.8.98.5 1.8.98.6] [RN:R11928 R11931 R11943 R11944]	Methanogenesis, methanol => methane	C1-methane	0	0	0	0	0	0	0	0	0
	K04480	methanol---5-hydroxybenzimidazolylcobamide Co-methyltransferase [EC:2.1.1.90] [RN:R09098]	Methanogenesis, methanol => methane	C1-methane	0	0	0	0	0	0	0	0	0
	K08264	heterodisulfide reductase [EC:1.8.98.1] [RN:R04540]	Methanogenesis, methanol => methane	C1-methane	0	0	0	0	0	0	0	0	0
	K08265	heterodisulfide reductase [EC:1.8.98.1] [RN:R04540]	Methanogenesis, methanol => methane	C1-methane	0	0	0	0	0	0	0	0	0
	K14080	[methyl-Co(III) methanol-specific corrinoid protein]:coenzyme M methyltransferase [EC:2.1.1.246] [RN:R09098]	Methanogenesis, methanol => methane	C1-methane	0	0	0	0	0	0	0	0	0
	K14081	methanol corrinoid protein [RN:R09098]	Methanogenesis, methanol => methane	C1-methane	0	0	0	0	0	0	0	0	0
	K14126	F420-non-reducing hydrogenase [EC:1.8.98.5] [RN:R11943]	Methanogenesis, methanol => methane	C1-methane	0	0	0	0	0	0	0	0	0
	K14127	F420-non-reducing hydrogenase iron-sulfur subunit [EC:1.8.98.5 1.8.98.6] [RN:R11943 R11944]	Methanogenesis, methanol => methane	C1-methane	0	0	0	0	0	0	0	0	0
	K14128	F420-non-reducing hydrogenase [EC:1.8.98.5] [RN:R11943]	Methanogenesis, methanol => methane	C1-methane	0	0	0	0	0	0	0	0	0
	K22480	heterodisulfide reductase 1 [EC:1.8.7.3] [RN:R11931]	Methanogenesis, methanol => methane	C1-methane	0	0	0	0	0	0	0	0	0
	K22481	heterodisulfide reductase 1 [EC:1.8.7.3] [RN:R11931]	Methanogenesis, methanol => methane	C1-methane	0	0	0	0	0	0	0	0	0
	K22482	heterodisulfide reductase 1 [EC:1.8.7.3] [RN:R11931]	Methanogenesis, methanol => methane	C1-methane	0	0	0	0	0	0	0	0	0
	K22516	formate dehydrogenase (coenzyme F420) [EC:1.8.98.6] [RN:R11944]	Methanogenesis, methanol => methane	C1-methane	0	0	0	0	0	0	0	0	0
	K00125	formate dehydrogenase (coenzyme F420) [EC:1.8.98.6] [RN:R11944]	Methanogenesis, methylamine/dimethylamine/trimethylamine => methane	C1-methane	0	0	0	0	0	0	0	0	0
	K00399	methyl-coenzyme M reductase [EC:2.8.4.1] [RN:R04541]	Methanogenesis, methylamine/dimethylamine/trimethylamine => methane	C1-methane	0	0	0	0	0	0	0	0	0
	K00401	methyl-coenzyme M reductase [EC:2.8.4.1] [RN:R04541]	Methanogenesis, methylamine/dimethylamine/trimethylamine => methane	C1-methane	0	0	0	0	0	0	0	0	0
	K00402	methyl-coenzyme M reductase [EC:2.8.4.1] [RN:R04541]	Methanogenesis, methylamine/dimethylamine/trimethylamine => methane	C1-methane	0	0	0	0	0	0	0	0	0
	K03388	heterodisulfide reductase 2 [EC:1.8.7.3 1.8.98.4 1.8.98.5 1.8.98.6] [RN:R11928 R11931 R11943 R11944]	Methanogenesis, methylamine/dimethylamine/trimethylamine => methane	C1-methane	0	0	0	0	0	0	0	0	0
	K03389	heterodisulfide reductase 2 [EC:1.8.7.3 1.8.98.4 1.8.98.5 1.8.98.6] [RN:R11928 R11931 R11943 R11944]	Methanogenesis, methylamine/dimethylamine/trimethylamine => methane	C1-methane	0	0	0	0	0	0	0	0	0
	K03390	heterodisulfide reductase 2 [EC:1.8.7.3 1.8.98.4 1.8.98.5 1.8.98.6] [RN:R11928 R11931 R11943 R11944]	Methanogenesis, methylamine/dimethylamine/trimethylamine => methane	C1-methane	0	0	0	0	0	0	0	0	0
	K08264	heterodisulfide reductase [EC:1.8.98.1] [RN:R04540]	Methanogenesis, methylamine/dimethylamine/trimethylamine => methane	C1-methane	0	0	0	0	0	0	0	0	0
	K08265	heterodisulfide reductase [EC:1.8.98.1] [RN:R04540]	Methanogenesis, methylamine/dimethylamine/trimethylamine => methane	C1-methane	0	0	0	0	0	0	0	0	0
	K14082	mtbA; [methyl-Co(III) methylamine-specific corrinoid protein]:coenzyme M methyltransferase [EC:2.1.1.247] [RN:R09998]	Methanogenesis, methylamine/dimethylamine/trimethylamine => methane	C1-methane	0	0	0	0	0	0	0	0	0
	K14083	mttB; trimethylamine---corrinoid protein Co-methyltransferase [EC:2.1.1.250] [RN:R09124]	Methanogenesis, methylamine/dimethylamine/trimethylamine => methane	C1-methane	0	0	0	0	0	0	0	0	0
	K14084	mttC; trimethylamine corrinoid protein [RN:R09124]	Methanogenesis, methylamine/dimethylamine/trimethylamine => methane	C1-methane	0	0	0	0	0	0	0	0	0
	K14126	F420-non-reducing hydrogenase [EC:1.8.98.5] [RN:R11943]	Methanogenesis, methylamine/dimethylamine/trimethylamine => methane	C1-methane	0	0	0	0	0	0	0	0	0
	K14127	F420-non-reducing hydrogenase iron-sulfur subunit [EC:1.8.98.5 1.8.98.6] [RN:R11943 R11944]	Methanogenesis, methylamine/dimethylamine/trimethylamine => methane	C1-methane	0	0	0	0	0	0	0	0	0
	K14128	F420-non-reducing hydrogenase [EC:1.8.98.5] [RN:R11943]	Methanogenesis, methylamine/dimethylamine/trimethylamine => methane	C1-methane	0	0	0	0	0	0	0	0	0
	K16176	mtmB; methylamine---corrinoid protein Co-methyltransferase [EC:2.1.1.248] [RN:R09998]	Methanogenesis, methylamine/dimethylamine/trimethylamine => methane	C1-methane	0	0	0	0	0	0	0	0	0
	K16177	mtmC; monomethylamine corrinoid protein [RN:R09998]	Methanogenesis, methylamine/dimethylamine/trimethylamine => methane	C1-methane	0	0	0	0	0	0	0	0	0
	K16178	mtbB; dimethylamine---corrinoid protein Co-methyltransferase [EC:2.1.1.249] [RN:R09999]	Methanogenesis, methylamine/dimethylamine/trimethylamine => methane	C1-methane	0	0	0	0	0	0	0	0	0
	K16179	mtbC; dimethylamine corrinoid protein [RN:R09999]	Methanogenesis, methylamine/dimethylamine/trimethylamine => methane	C1-methane	0	0	0	0	0	0	0	0	0
	K22480	heterodisulfide reductase 1 [EC:1.8.7.3] [RN:R11931]	Methanogenesis, methylamine/dimethylamine/trimethylamine => methane	C1-methane	0	0	0	0	0	0	0	0	0
	K22481	heterodisulfide reductase 1 [EC:1.8.7.3] [RN:R11931]	Methanogenesis, methylamine/dimethylamine/trimethylamine => methane	C1-methane	0	0	0	0	0	0	0	0	0
	K22482	heterodisulfide reductase 1 [EC:1.8.7.3] [RN:R11931]	Methanogenesis, methylamine/dimethylamine/trimethylamine => methane	C1-methane	0	0	0	0	0	0	0	0	0
	K22516	formate dehydrogenase (coenzyme F420) [EC:1.8.98.6] [RN:R11944]	Methanogenesis, methylamine/dimethylamine/trimethylamine => methane	C1-methane	0	0	0	0	0	0	0	0	0
Acetogenesis	K00194	acetyl-CoA decarbonylase/synthase complex [EC:2.3.1.-] [RN:R10243]	Reductive acetyl-CoA pathway (Wood-Ljungdahl pathway)	C1	0	0	0	0	0	0	0	0	0
	K00197	acetyl-CoA decarbonylase/synthase complex [EC:2.3.1.-] [RN:R10243]	Reductive acetyl-CoA pathway (Wood-Ljungdahl pathway)	C1	0	0	0	0	0	0	0	0	0
	K00198	carbon-monoxide dehydrogenase [EC:1.2.7.4] [RN:R07157]	Reductive acetyl-CoA pathway (Wood-Ljungdahl pathway)	C1	0	0	0	0	0	0	0	0	0
	K00297	methylenetetrahydrofolate reductase (NADPH) [EC:1.5.1.20] [RN:R07168]	Reductive acetyl-CoA pathway (Wood-Ljungdahl pathway)	C1	0	1	1	1	0	1	0	1	1
	K01491	methylenetetrahydrofolate dehydrogenase (NADP+) / methenyltetrahydrofolate cyclohydrolase [EC:1.5.1.5 3.5.4.9] [RN:R01655 R01220]	Reductive acetyl-CoA pathway (Wood-Ljungdahl pathway)	C1	0	1	1	1	0	1	1	1	1
	K01938	formate--tetrahydrofolate ligase [EC:6.3.4.3] [RN:R00943]	Reductive acetyl-CoA pathway (Wood-Ljungdahl pathway)	C1	0	0	0	1	0	1	0	0	1
	K05299	formate dehydrogenase [EC:1.17.1.10] [RN:R00134]	Reductive acetyl-CoA pathway (Wood-Ljungdahl pathway)	C1	0	0	0	0	0	0	0	0	0
	K14138	acetyl-CoA synthase [EC:2.3.1.169] [RN:R08433]	Reductive acetyl-CoA pathway (Wood-Ljungdahl pathway)	C1	0	0	0	0	0	0	0	0	0
	K15022	formate dehydrogenase [EC:1.17.1.10] [RN:R00134]	Reductive acetyl-CoA pathway (Wood-Ljungdahl pathway)	C1	0	0	0	0	0	0	0	0	0
	K15023	5-methyltetrahydrofolate corrinoid/iron sulfur protein methyltransferase [EC:2.1.1.258] [RN:R02289]	Reductive acetyl-CoA pathway (Wood-Ljungdahl pathway)	C1	0	0	0	0	0	0	0	0	0
Sulfate Reduction	K00380	sulfite reductase (NADPH) [EC:1.8.1.2] [RN:R00858]	Assimilatory sulfate reduction, sulfate => H2S	Sulfur	0	0	0	0	0	0	0	0	0
	K00381	sulfite reductase (NADPH) [EC:1.8.1.2] [RN:R00858]	Assimilatory sulfate reduction, sulfate => H2S	Sulfur	0	1	1	0	0	1	1	0	0
	K00390	phosphoadenosine phosphosulfate reductase [EC:1.8.4.8] [RN:R02021]	Assimilatory sulfate reduction, sulfate => H2S	Sulfur	0	1	1	0	0	1	1	1	0
	K00392	sulfite reductase (ferredoxin) [EC:1.8.7.1] [RN:R00859]	Assimilatory sulfate reduction, sulfate => H2S	Sulfur	0	0	0	0	0	0	0	0	0
	K00860	adenylylsulfate kinase [EC:2.7.1.25] [RN:R00509]	Assimilatory sulfate reduction, sulfate => H2S	Sulfur	0	0	0	0	0	0	0	0	0
	K00955	sulfate adenylyltransferase [EC:2.7.7.4] [RN:R00529]	Assimilatory sulfate reduction, sulfate => H2S	Sulfur	0	1	0	0	0	0	1	1	0
	K00956	sulfate adenylyltransferase [EC:2.7.7.4] [RN:R00529]	Assimilatory sulfate reduction, sulfate => H2S	Sulfur	0	0	1	0	0	0	0	0	0
	K00957	sulfate adenylyltransferase [EC:2.7.7.4] [RN:R00529]	Assimilatory sulfate reduction, sulfate => H2S	Sulfur	0	1	1	0	0	0	1	1	0
	K00958	sulfate adenylyltransferase [EC:2.7.7.4] [RN:R00529]	Assimilatory sulfate reduction, sulfate => H2S	Sulfur	0	0	0	0	0	1	0	0	0
	K13811	sulfate adenylyltransferase [EC:2.7.7.4] [RN:R00529]	Assimilatory sulfate reduction, sulfate => H2S	Sulfur	0	0	0	0	0	0	0	0	0
	K00394	adenylylsulfate reductase [EC:1.8.99.2] [RN:R08553]	Dissimilatory sulfate reduction, sulfate => H2S	Sulfur	0	0	0	0	0	0	0	0	0
	K00395	adenylylsulfate reductase [EC:1.8.99.2] [RN:R08553]	Dissimilatory sulfate reduction, sulfate => H2S	Sulfur	0	0	0	0	0	0	0	0	0
	K00956	sulfate adenylyltransferase [EC:2.7.7.4] [RN:R00529]	Dissimilatory sulfate reduction, sulfate => H2S	Sulfur	0	0	1	0	0	0	0	0	0
	K00957	sulfate adenylyltransferase [EC:2.7.7.4] [RN:R00529]	Dissimilatory sulfate reduction, sulfate => H2S	Sulfur	0	1	1	0	0	0	1	1	0
	K00958	sulfate adenylyltransferase [EC:2.7.7.4] [RN:R00529]	Dissimilatory sulfate reduction, sulfate => H2S	Sulfur	0	0	0	0	0	1	0	0	0
	K11180	sulfite reductase, dissimilatory-type [EC:1.8.99.5] [RN:R00861]	Dissimilatory sulfate reduction, sulfate => H2S	Sulfur	0	0	0	0	0	0	0	0	0
	K11181	sulfite reductase, dissimilatory-type [EC:1.8.99.5] [RN:R00861]	Dissimilatory sulfate reduction, sulfate => H2S	Sulfur	0	0	0	0	0	0	0	0	0
	K17222	soxA; sulfur-oxidizing protein SoxA	Thiosulfate oxidation by SOX complex, thiosulfate => sulfate	Sulfur	0	0	0	0	0	0	0	0	0
	K17223	soxX; sulfur-oxidizing protein SoxX	Thiosulfate oxidation by SOX complex, thiosulfate => sulfate	Sulfur	0	0	0	0	0	0	0	0	0
	K17224	soxB; sulfur-oxidizing protein SoxB	Thiosulfate oxidation by SOX complex, thiosulfate => sulfate	Sulfur	0	0	0	0	0	0	0	0	0
	K17225	soxC; S-disulfanyl-L-cysteine oxidoreductase SoxC [EC:1.8.2.6]	Thiosulfate oxidation by SOX complex, thiosulfate => sulfate	Sulfur	0	0	0	0	0	0	0	0	0
	K17226	soxY; sulfur-oxidizing protein SoxY	Thiosulfate oxidation by SOX complex, thiosulfate => sulfate	Sulfur	0	1	0	0	0	0	0	0	0
	K17227	soxZ; sulfur-oxidizing protein SoxZ	Thiosulfate oxidation by SOX complex, thiosulfate => sulfate	Sulfur	0	0	0	0	0	0	0	0	0
	K22622	soxD; S-disulfanyl-L-cysteine oxidoreductase SoxD [EC:1.8.2.6]	Thiosulfate oxidation by SOX complex, thiosulfate => sulfate	Sulfur	0	0	0	0	0	0	0	0	0
	K01011	rhodanase		Sulfur	1	1	1	0	0	1	1	1	0
	K08352	thiosulfate reductase / polysulfide reductase chain A [EC:1.8.5.5]		Sulfur	0	0	0	0	0	0	0	0	0
	K08357	tetrathionate => thiosulfate		Sulfur	0	0	0	0	0	0	0	0	0
Denitrification	K00368	nitrite reductase (NO-forming) [EC:1.7.2.1] [RN:R00783 R00785]	Denitrification, nitrate => nitrogen	Nitrogen	0	0	0	0	0	0	0	0	0
	K00370	nitrate reductase 1 [EC:1.7.99.-] [RN:R00798]	Denitrification, nitrate => nitrogen	Nitrogen	0	1	0	0	0	0	0	0	0
	K00371	nitrate reductase 1 [EC:1.7.99.-] [RN:R00798]	Denitrification, nitrate => nitrogen	Nitrogen	0	1	0	0	0	0	0	0	0
	K00374	nitrate reductase 1 [EC:1.7.99.-] [RN:R00798]	Denitrification, nitrate => nitrogen	Nitrogen	0	1	0	0	0	0	0	0	0
	K00376	nitrous-oxide reductase [EC:1.7.2.4] [RN:R02804]	Denitrification, nitrate => nitrogen	Nitrogen	0	0	0	0	0	0	0	0	0
	K02305	nitric oxide reductase [EC:1.7.2.5] [RN:R00294]	Denitrification, nitrate => nitrogen	Nitrogen	0	0	0	0	0	0	0	0	0
	K02567	periplasmic nitrate reductase NapA [EC:1.7.99.-] [RN:R00798]	Denitrification, nitrate => nitrogen	Nitrogen	0	0	0	0	0	0	0	0	0
	K02568	cytochrome c-type protein NapB [RN:R00798]	Denitrification, nitrate => nitrogen	Nitrogen	0	0	0	0	0	0	0	0	0
	K04561	nitric oxide reductase [EC:1.7.2.5] [RN:R00294]	Denitrification, nitrate => nitrogen	Nitrogen	0	0	0	0	0	0	0	0	0
	K15864	nitrite reductase (NO-forming) [EC:1.7.2.1] [RN:R00783 R00785]	Denitrification, nitrate => nitrogen	Nitrogen	0	0	0	0	0	0	0	0	0
	K15877	fungal nitric oxide reductase [EC:1.7.1.14] [RN:R02492 R09446 R09808 R09809]	Denitrification, nitrate => nitrogen	Nitrogen	0	0	0	0	0	0	0	0	0
Iron Transport and Reduction	AA8	AA8 Iron reductase domain	Auxiliary Activities	CAZY	0	0	0	0	0	0	0	0	0
	K02010	ABC.FE.A; iron(III) transport system ATP-binding protein [EC:3.6.3.30]	Iron(III) transport system		0	0	1	0	0	0	0	0	0
	K02011	ABC.FE.P; iron(III) transport system permease protein	Iron(III) transport system		0	1	3	0	0	2	0	0	0
	K02012	ABC.FE.S; iron(III) transport system substrate-binding protein	Iron(III) transport system		0	1	3	0	0	2	0	0	0
	K02013	ABC.FEV.A; iron complex transport system ATP-binding protein [EC:3.6.3.34]	Iron complex transport system		0	1	1	1	1	3	1	1	0
	K02015	ABC.FEV.P; iron complex transport system permease protein	Iron complex transport system		0	0	0	0	0	2	0	0	0
	K02016	ABC.FEV.S; iron complex transport system substrate-binding protein	Iron complex transport system		0	0	0	0	0	1	0	0	0
	K09818	ABC.MN.S; manganese/iron transport system substrate-binding protein	Manganese/iron transport system		0	0	0	0	0	0	0	0	0
	K09819	ABC.MN.P; manganese/iron transport system permease protein	Manganese/iron transport system		0	0	0	0	0	0	0	0	0
	K09820	ABC.MN.A; manganese/iron transport system ATP-binding protein	Manganese/iron transport system		0	0	0	0	0	0	0	0	0
	K10616	cymAa; p-cymene methyl-monooxygenase [EC:1.14.15.25] [RN:R05266]	Cymene degradation, p-cymene => p-cumate	hydrocarbon degradation	0	0	0	0	0	0	0	0	0
	K10617	cymB; p-cumic alcohol dehydrogenase [RN:R05232]	Cymene degradation, p-cymene => p-cumate	hydrocarbon degradation	0	0	0	0	0	0	0	0	0
	K10618	cymC; p-cumic aldehyde dehydrogenase [RN:R05235]	Cymene degradation, p-cymene => p-cumate	hydrocarbon degradation	0	0	0	0	0	0	0	0	0
	K11604	sitA; manganese/iron transport system substrate-binding protein	Manganese/iron transport system		0	0	0	0	0	0	0	0	0
	K11605	sitC; manganese/iron transport system permease protein	Manganese/iron transport system		0	0	0	0	0	1	0	0	0
	K11606	sitD; manganese/iron transport system permease protein	Manganese/iron transport system		0	0	0	0	0	0	0	0	0
	K11607	sitB; manganese/iron transport system ATP-binding protein	Manganese/iron transport system		0	0	0	0	0	1	0	0	0
	K11704	mtsA; iron/zinc/manganese/copper transport system substrate-binding protein	Iron/zinc/manganese/copper transport system		0	0	0	0	0	0	0	0	0
	K11705	mtsC; iron/zinc/manganese/copper transport system permease protein	Iron/zinc/manganese/copper transport system		0	0	0	0	0	0	0	0	0
	K11706	mtsB; iron/zinc/manganese/copper transport system ATP-binding protein	Iron/zinc/manganese/copper transport system		0	0	0	0	0	0	0	0	0
	K11707	troA; manganese/zinc/iron transport system substrate-binding protein	Manganese/zinc/iron transport system		0	0	0	0	0	0	0	0	0
	K11708	troC; manganese/zinc/iron transport system permease protein	Manganese/zinc/iron transport system		0	0	0	0	0	0	0	0	0
	K11709	troD; manganese/zinc/iron transport system permease protein	Manganese/zinc/iron transport system		0	0	0	0	0	0	0	0	0
	K11710	troB; manganese/zinc/iron transport system ATP-binding protein	Manganese/zinc/iron transport system		0	0	0	0	0	0	0	0	0
	K18293	cymAb; p-cymene methyl-monooxygenase electron transfer component [EC:1.18.1.3]	Cymene degradation, p-cymene => p-cumate	hydrocarbon degradation	0	0	0	0	0	0	0	0	0

SI Table 6. Concentration of hydrogen and methane (ppm) and percent ratio for the second follow up reactor series (47 °C and ~1800 psi with a 15% H_2/85% CH_4 gas blend) at each time point.

			H_2 (ppm)	CH_4 (ppm)	H_2 (%)	CH_4 (%)
Gas Canister (15% H_2/85% CH_4)			28976	195534	12.91	87.09
Without sediment	Abiotic	Day 1	56687	462273	10.92	89.08
		Day 2	56688	476605	10.63	89.37
		Day 3	70258	539718	11.52	88.48
	Abiotic Replicate	Day 1	N/A	161762	0.00	100.00
		Day 2	70029	545742	11.37	88.63
		Day 3	60111	501851	10.70	89.30
	Biotic	Day 1	79781	558612	12.50	87.50
		Day 2	45569	423783	9.71	90.29
		Day 3	67895	511815	11.71	88.29
	Biotic Replicate	Day 1	43000	354139	10.83	89.17
		Day 2	60115	501788	10.70	89.30
		Day 3	57839	426418	11.94	88.06

SI Table 7. Ethane gas measurements for the initial reactor series (47 ℃ and ~1800 psi with a 15% H_2/85% CH_4 gas blend) at each time point.

			Ethane (ppm)
**With Sediment**	**Biotic**	**Day 1**	62
		**Day 2**	69
		**Day 7**	43
		**Day 21**	25
	**Biotic Replicate**	**Day 1**	12
		**Day 2**	75
		**Day 7**	71
		**Day 21**	67

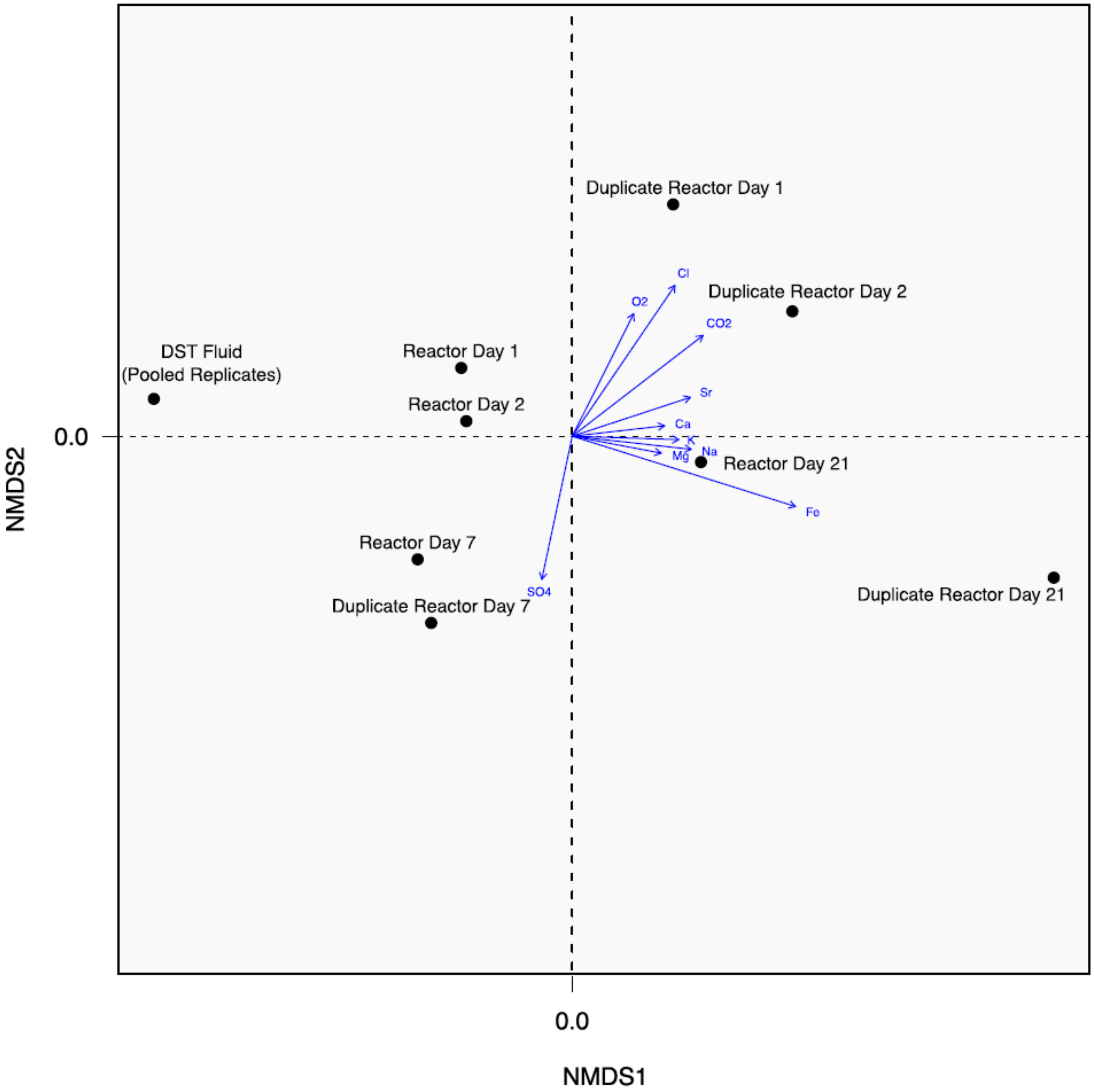

SI Figure 3. Non-metric multidimensional scaling (NMDS) plot of samples with 3 dimensions and a stress value of 0.0241. This plot was constructed using Bray-Curtis distance calculated after sequence libraries were resampled to the depth of the sample with the fewest sequences from this experiment (1046 sequences per sample). Environmental vectors for geochemical parameters were fitted onto the ordination plot, with the direction of the arrow corresponding to the direction of the gradient and the length of the vector proportional to the strength of the correlation between ordination and environmental variables. The only statistically significant vector was Cl (R^2=0.7941, p-val=0.033) with no significant correlation measured using the Mantel test. Vector information for all other environmental vectors is in SI Table 8.

SI Table 8. R^2 and p-values for all constructed environmental vectors on the NMDS plot (Figure 2).

	R^2	p-val
B	0.1794	0.708
Ba	0.0314	0.971
Ca	0.1253	0.800
Fe	0.8565	0.166
K	0.1152	0.795
Li	0.3125	0.465
Mg	0.1457	0.774
Mn	0.0895	0.757
Na	0.2084	0.633
Sr	0.1694	0.704
Cl	0.7941	0.033*
SO_4	0.5910	0.158

*p-value ≤ 0.05
